# Clinical, neuroradiological, and molecular characterization of mitochondrial threonyl-tRNA-synthetase (TARS2)-related disorder

**DOI:** 10.1016/j.gim.2023.100938

**Published:** 2023-11

**Authors:** Andrea Accogli, Sheng-Jia Lin, Mariasavina Severino, Sung-Hoon Kim, Kevin Huang, Clarissa Rocca, Megan Landsverk, Maha S. Zaki, Almundher Al-Maawali, Varunvenkat M. Srinivasan, Khalid Al-Thihli, G. Bradly Schaefer, Monica Davis, Davide Tonduti, Chiara Doneda, Lara M. Marten, Chris Mühlhausen, Maria Gomez, Eleonora Lamantea, Rafael Mena, Mathilde Nizon, Vincent Procaccio, Amber Begtrup, Aida Telegrafi, Hong Cui, Heidi L. Schulz, Julia Mohr, Saskia Biskup, Mariana Amina Loos, Hilda Verónica Aráoz, Vincenzo Salpietro, Laura Davis Keppen, Manali Chitre, Cassidy Petree, Lucy Raymond, Julie Vogt, Lindsey B. Sawyer, Alice A. Basinger, Signe Vandal Pedersen, Toni S. Pearson, Dorothy K. Grange, Lokesh Lingappa, Paige McDunnah, Rita Horvath, Benjamin Cognè, Bertrand Isidor, Andreas Hahn, Karen W. Gripp, Seyed Mehdi Jafarnejad, Elsebet Østergaard, Carlos E. Prada, Daniele Ghezzi, Vykuntaraju K. Gowda, Robert W. Taylor, Nahum Sonenberg, Henry Houlden, Marie Sissler, Gaurav K. Varshney, Reza Maroofian

**Affiliations:** 1 Division of Medical Genetics, Department of Specialized Medicine, Montreal Children's Hospital, McGill University Health Centre (MUHC), Montreal, Canada; 2 Department of Human Genetics, McGill University, Montreal, QC, Canada; 3 Genes & Human Disease Research Program, Oklahoma Medical Research Foundation, Oklahoma City, OK; 4 Neuroradiology Unit, IRCCS Istituto Giannina Gaslini, Genoa, Italy; 5 Goodman Cancer Institute, McGill University, Montreal, Canada; 6 Department of Biochemistry, McGill University, Montreal, Canada; 7 Department of Neuromuscular Diseases, UCL Queen Square Institute of Neurology, London, United Kingdom; 8 University of South Dakota Sanford School of Medicine Sioux Falls, SD; 9 Sanford Research, Pediatrics and Rare Diseases Group, Sioux Falls, SD; 10 Human Genetics and Genome Research Institute, Clinical Genetics Department, National Research Centre, Cairo, Egypt; 11 Department of Genetics, College of Medicine and Health Sciences, Sultan Qaboos University, Muscat, Oman; 12 Genetic and Developmental Medicine Clinic, Sultan Qaboos University Hospital, Muscat, Oman; 13 Indira Gandhi Institute of Child Health, Bangalore, India; 14 Department of Pediatrics, University of Arkansas for Medical Sciences, Little Rock, AR; 15 Unit of Pediatric Neurology, COALA (Center for Diagnosis and Treatment of Leukodystrophies), V. Buzzi Children's Hospital, Milan, Italy; 16 Department of Biomedical and Clinical Sciences, University of Milan, Milan, Italy; 17 Pediatric Radiology and Neuroradiology Department, Children's Hospital Vittore Buzzi, Milan, Italy; 18 Department of Pediatrics and Adolescent Medicine, University Medical Center Göttingen, Germany; 19 Centro de Obsetricia y Ginecologia & Centro Medico Moderno, Santo Domingo, Dominican Republic; 20 Unit of Medical Genetics and Neurogenetics, Fondazione IRCCS Istituto Neurologico Carlo Besta, Milan, Italy; 21 Division of Neonatology, Cincinnati Children's Hospital Medical Center, Cincinnati, OH; 22 Centro de Obsetricia y Ginecologia, Santo Domingo, Dominican Republic; 23 Service de Génétique Médicale, CHU de Nantes, Nantes Université, Nantes, France; 24 Nantes Université, CNRS, INSERM, l'Institut du Thorax, Nantes, France; 25 University of Angers, MitoLab Team, Unité MitoVasc, UMR CNRS 6015, INSERM U1083, SFR ICAT, Angers, France; 26 Department of Genetics, CHU Angers, Angers, France; 27 GeneDx, Gaithersburg, MD; 28 Human Genetic center Tübingen, Baden-Württemberg, Germany; 29 CeGaT GmbH, Germany; 30 Department of Neurology, Hospital de Pediatría Juan P. Garrahan, Buenos Aires, Argentina; 31 Genomics Laboratory, Hospital de Pediatría Juan P. Garrahan, Buenos Aires, Argentina; 32 Department of Biotechnological and Applied Clinical Sciences, University of L'Aquila, L'Aquila, Italy; 33 Cambridge University Hospitals NHS Foundation Trust, Cambridge, United Kingdom; 34 West Midlands Regional Genetics Service, Birmingham Women and Children's Hospital NHS Foundation Trust, Birmingham, United Kingdom; 35 Children’s Hospital of the King’s Daughters, Norfolk, Virginia, VA; 36 Department of Genetics, Copenhagen University Hospital Rigshospitalet, Copenhagen, Denmark; 37 Department of Neurology, Washington University School of Medicine, St. Louis, MO; 38 Division of Genetics and Genomic Medicine, Department of Pediatrics, Washington University School of Medicine, St. Louis, MO; 39 Center for the Investigation of Membrane Excitability Diseases (CIMED), St. Louis, MO; 40 Rainbow Children Hospital, Hyderabad, India; 41 Division of Medical Genetics, Nemours/A I duPont Hospital for Children, Wilmington, DE; 42 Department of Clinical Neurosciences, University of Cambridge, Cambridge, United Kingdom; 43 Service de Génétique Médicale, CHU de Nantes, Nantes Université, Nantes, France; 44 Nantes Université, CNRS, INSERM, l'Institut du Thorax, Nantes, France; 45 Department of Child Neurology, University Hospital, Gießen, Germany; 46 Patrick G. Johnston Centre for Cancer Research, Queen’s University Belfast, Belfast, United Kingdom; 47 Department of Clinical Medicine, University of Copenhagen, Copenhagen, Denmark; 48 Division of Genetics, Genomics, and Metabolism, Ann & Robert Lurie Children's Hospital of Chicago, Chicago; 49 Department of Pediatrics, Feinberg School of Medicine, Northwestern University, Chicago; 50 Fundacion Cardiovascular de Colombia, Floridablanca, Colombia; 51 Department of Pathophysiology and Transplantation, University of Milan, Milan, Italy; 52 Wellcome Centre for Mitochondrial Research, Translational and Clinical Research Institute, Newcastle University, Newcastle upon Tyne, United Kingdom; 53 NHS Highly Specialized Service for Rare Mitochondrial Disorders of Adults and Children, Newcastle University, Newcastle upon Tyne, United Kingdom; 54 ARNA - UMR5320 CNRS - U1212 INSERM, Université de Bordeaux, IECB, Pessac, France

**Keywords:** Cerebellar atrophy, Mitochondrial dysfunction, Mitochondrial threonyl-tRNA-synthetase, mTORC1 signaling, *TARS2*

## Abstract

**Purpose:**

Biallelic variants in *TARS2*, encoding the mitochondrial threonyl-tRNA-synthetase, have been reported in a small group of individuals displaying a neurodevelopmental phenotype but with limited neuroradiological data and insufficient evidence for causality of the variants.

**Methods:**

Exome or genome sequencing was carried out in 15 families. Clinical and neuroradiological evaluation was performed for all affected individuals, including review of 10 previously reported individuals. The pathogenicity of *TARS2* variants was evaluated using in vitro assays and a zebrafish model.

**Results:**

We report 18 new individuals harboring biallelic *TARS2* variants. Phenotypically, these individuals show developmental delay/intellectual disability, regression, cerebellar and cerebral atrophy, basal ganglia signal alterations, hypotonia, cerebellar signs, and increased blood lactate. In vitro studies showed that variants within the TARS2^301-381^ region had decreased binding to Rag GTPases, likely impairing mTORC1 activity. The zebrafish model recapitulated key features of the human phenotype and unraveled dysregulation of downstream targets of mTORC1 signaling. Functional testing of the variants confirmed the pathogenicity in a zebrafish model.

**Conclusion:**

We define the clinico-radiological spectrum of *TARS2*-related mitochondrial disease, unveil the likely involvement of the mTORC1 signaling pathway as a distinct molecular mechanism, and establish a *TARS2* zebrafish model as an important tool to study variant pathogenicity.

## Graphical abstract

**Figure:**
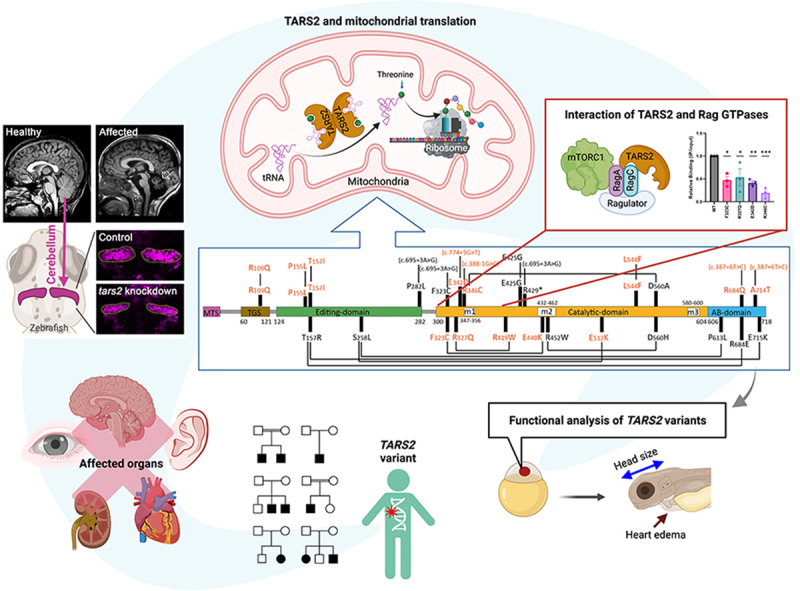

## Introduction

Aminoacyl-transfer RNA synthetases (aaRSs) are a family of nuclear-encoded enzymes catalyzing the binding of specific amino acids to their corresponding transfer RNAs (tRNAs), a crucial step for precise protein synthesis from genetic information. These enzymes are classified in 2 groups: cytosolic aaRSs (also named ARSs), serving in the cytoplasm for messenger RNA (mRNA) translation and mitochondrial mt-aaRSs (also known as ARS2s), which charge mitochondrial tRNA with amino acids after being translated by cytosolic ribosomes and transferred into the mitochondrial matrix.

ARSs and ARS2s are encoded by distinct nuclear genes, with the exception of the glycyl-and lysyl-tRNA synthetase genes (*GARS1* and *KARS1*, respectively) that encode 2 isoforms with cytosolic and mitochondrial localization.^1^ ARS2s, along with other translation factors, are thus critical to ensure the correct translation of mitochondrial DNA and, in turn, the assembly and function of mitochondrial oxidative phosphorylation complexes containing mitochondrial DNA–encoded subunits (cI, cIII, cIV, and cV). Dysfunction in any component of the mitochondrial translation machinery can therefore affect oxidative phosphorylation assembly and stability, resulting in combined enzyme deficiencies.^2^ Indeed, pathogenic variants in all 17 ARS2 (plus GARS1 and KARS1) genes lead to mitochondrial dysfunction that results in a variety of pediatric and adult-onset disorders presenting as encephalopathy, leukodystrophy, neuropathy, cardiomyopathy, sensorineural hearing loss, and endocrine dysfunction.^3^, ^4^, ^5^, ^6^ Besides impaired aminoacylation, other factors have been reported to explain the variety of phenotypes present in ARS2-related disorders. These factors include non-canonical functions, tissue-specific developmental differences in energy requirements, and downstream effects of mitochondrial dysfunction on other intracellular organelles.^7^^,^^8^

Biallelic variants in *TARS2*, encoding the mitochondrial threonyl-tRNA-synthetase, have been previously associated with a neurodevelopmental disorder (NDD) called combined oxidative phosphorylation deficiency 21 (MIM#615918), characterized by developmental delay and regression, microcephaly, variable brain abnormalities, and epilepsy in 10 individuals with either limited clinical and neuroradiological data or insufficient data supporting causality of the variants.^9^, ^10^, ^11^ In addition to the canonical aminoacylation function, a recent study has unveiled a non-canonical role for TARS2 in the regulation of mTORC1 activity in response to changing levels of the amino acid threonine.^12^

Here, we present 18 new individuals from 15 different families harboring biallelic *TARS2* variants and neuroradiological findings from 5 recently reported individuals with *TARS2* variants,^11^ outlining the molecular and phenotypic spectrum of *TARS2*-related mitochondrial disease. Moreover, with in vitro studies, we discover that some *TARS2* variants disrupt mTORC1 signaling and generate a zebrafish model that recapitulates human phenotypes and supports the functional testing of novel or poorly characterized *TARS2* variants.

## Material and Methods

### Genetic analysis of affected individuals

**Figure 1:**
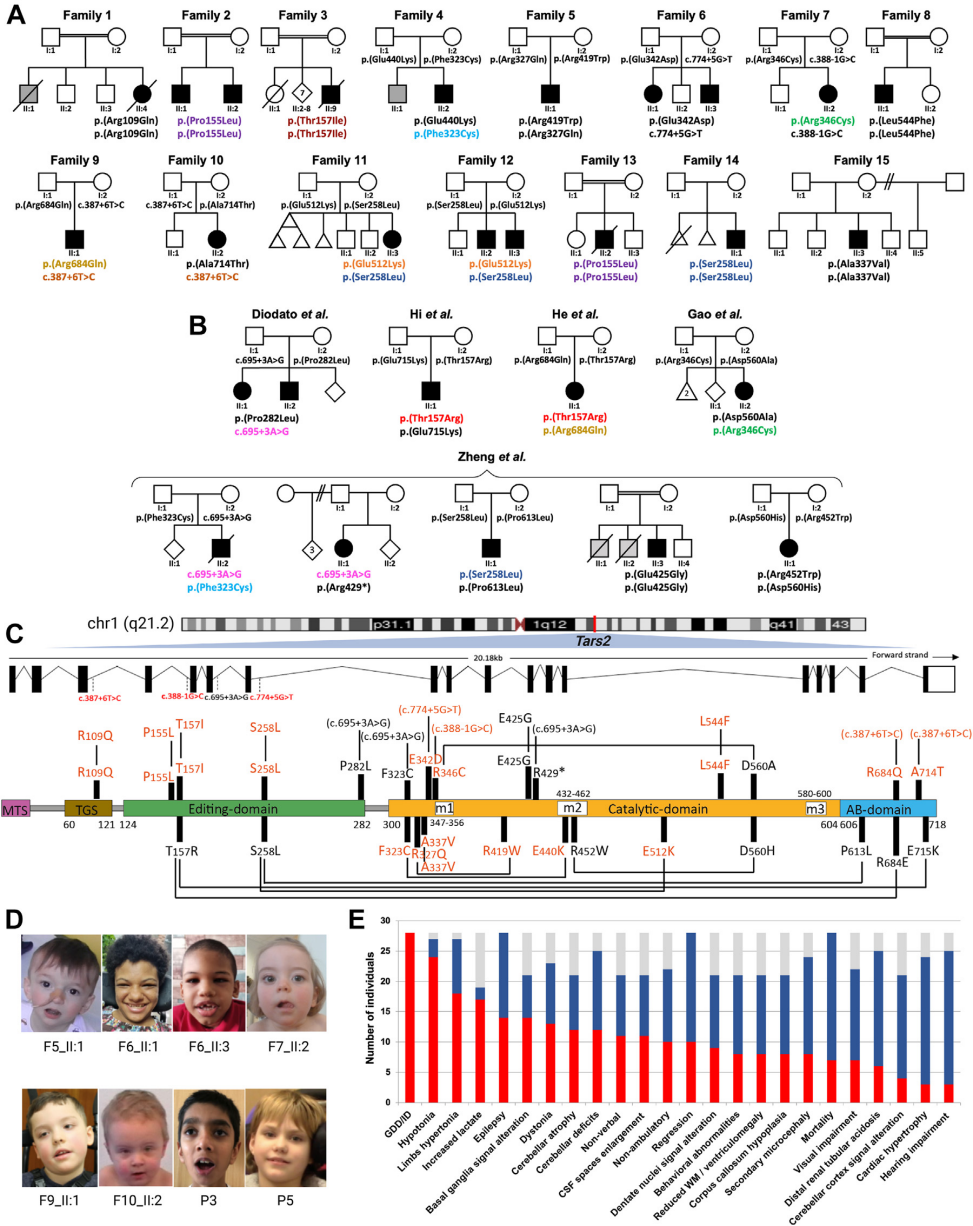
**Clinical summary of individuals with biallelic *TARS2* variants.** Pedigree of families 1-15 (A) and previously reported individuals (B). Genotypes of tested individuals are indicated under the symbols. Variants observed in more than 1 family have been shown in different colors. In the pedigree, squares represent males, circles represent females, black shaded symbols denote affected individuals harboring biallelic *TARS2* variants, and the gray shaded symbol refer to individuals presumably affected by the same disorder yet not tested. C. Schematic depiction of *TARS2* transcript (ENSG00000143374) and the modular human mitochondrial threonyl-tRNA synthetase (TARS2) protein (ENSP00000358060) with localization of the disease associated variants. The different functional domains are shown to scale, named, and colored. MTS and AB stand for mitochondrial targeting sequence and anticodon-binding, respectively. m1, m2, and m3 are catalytic motifs 1, 2, and 3, respectively. Missense variants are indicated in red for those reported in the present study and in black for those reported elsewhere. Allelic compositions, as identified in individuals, are linked through black lines. Variants that do not lead to missense variants are indicated in brackets (because they correspond to genomic modifications). Although amino acid conversion of a given mutation is generally preceded by the letter “p.,” this is omitted for sake of simplicity. D. Clinical features of individuals with biallelic *TARS2* variants. Subtle and non-specific dysmorphic features are indicated, such as thin upper lip vermilion in individual II:1 of family 5; mild coarse facial features in individuals II:1 and II:3 of family 6; short nose, long philtrum, and thin upper lip vermilion in individual II:2 of family 7; left eye strabismus and right uplifted earlobe in individual II:1 of family 9; broad and prominent forehead, sparse eyebrows, infraorbital creases, thin upper lip vermilion, and dimple chin in patient II:2 of family 10; thick eyebrows in individual 3; and deep set eyes in individual 5 previously reported by Zheng et al.^11^^,^^12^ E. Phenotypic bar graph showing the most relevant clinical and radiological features among all individuals so far identified with biallelic *TARS2* variants. Red: number of individuals out of 28 showing each feature. Blue: number of individuals without each specific feature. Gray: number of individuals for whom a specific feature or the brain MRI was not available.

Seventeen previously unreported individuals from 15 unrelated families of various ancestries (European, Hispanic, Chinese, South Asian, Middle Eastern, and North African) were included in this study after written informed consent was obtained from the parents (Figure 1A, Supplemental Table 2). Detailed clinical features and family histories were recorded for all individuals. Developmental outcome was defined by each treating clinician as mild, moderate, severe, or profound intellectual disability for individuals >5 years old or developmental delay for individuals <5 years old, based on age-appropriate metrics. When possible formal assessment for intellecual disability was performed in older children assessing QI range as follows: 50 to 69 mild, 35 to 49 moderate, 20 to 34 severe, and <20 profound. Brain magnetic resonance imaging (MRI) studies were available for review in 10 of 17 individuals of the present cohort and in 5 additional cases recently included in another study.^11^ The first brain MRI was performed at an average age of 2.8 years (range: 1 month-13.5 years). Follow-up brain MRI studies were available in 7 individuals (mean follow-up duration of 2.8 years, range: 1 month-5 years). Overall, 25 brain MRI scans were reviewed in consensus by 2 pediatric neuroradiologists (M.S. and C.D.).

Exome or genome sequencing was performed in probands in the respective collaborating centers using different analysis platforms according to the Burrows-Wheeler Aligner/The Genome Analysis Tool kit–based pipeline. Sanger sequencing with standard methods was performed for candidate variant validation and familial segregation. All *TARS2* variants are reported according to the NM_025150.5 transcript and classified according to the American College of Medical Genetics and Genomics and the Association for Molecular Pathology standards and guidelines.^13^

### Structural analysis of TARS2 variants

The structural environment of the residues affected by missense variations in individuals was investigated using 3 structural templates: dimeric threonyl-tRNA synthetase from *Staphylococcus aureus* (PDB: 1NYR),^14^ 1 monomer of the *Escherichia coli* threonyl-tRNA synthetase, in complex with cognate tRNA with the CCA extremity of the tRNA pointing into the catalytic core (PDB: 1QF6),^15^ or a 3D model of the human TARS2 obtained within misynpat.org.^16^ A multiple sequence alignment makes it possible to identify the correspondence of the residues in the 3 templates (Supplemental Figure 1).

### Functional Studies

Methods related to in vitro and in vivo functional studies are described in Supplemental methods.

## Results

### Genetic findings

We identified 16 novel or ultra-rare *TARS2* variants in either a homozygous (*n* = 7) or compound heterozygous state (*n* = 10) (Figure 1A, Table 1). Seven homozygous missense variants were identified in families 1, 2, 3, 8, 13, 14, and 15. With the exception of a patient from family 5 harboring compound heterozygous missense variants, other affected members of families 6, 7, 9, and 10 were carrying missense variants in a compound heterozygous state with splice site variants (Figure 1A). Sanger sequencing confirmed segregation of the variants with the phenotype within these families. Remarkably, variants c.464C>T p.(Pro155Leu), c.1534G>A p.(Glu512Lys), and c.773C>T p.(Ser258Leu) were recurrent in the present cohort (families 2,11, 12, 13, and 14) (Figure 1A). The latter, along with the variants c.470G>C p.(Thr157Arg), c.968T>G p.(Phe323Cys), and c.1036C>T p.(Arg346Cys) have been also found in compound heterozygous states in other reported individuals (Figure 1B) with *TARS2-*related disorder.^17^^,^^18^ The position of each variant is depicted in Figure 1C.

**Table 1:** Genetic and phenotypic features of subjects with TARS2 variants

	Family 1	Family 2		Family 3	Family 4	Family 5	Family 6		Family 7	Family 8	Family 9	Family 10	Family 11	Family 12		Family 13	Family 14	Family 15	Zheng et al. 2022					He et al. 2022	Gao et al. 2022	Li et al. 2020	Diodato et al. 2014		Summary
**Subject**	II:4	II:1	II:2	II:9	II:2	II:1	II:1	II:3	II:2	II:1	II:1	II:2	II:3	II:2	II:3	II:2	II:1	II:3	F11S1	F12S1	F13S1	F14S1	F15S1				P2	P3	
***TARS2* variants (NM_025150.5)**	c.326G>A p.(Arg109Gln)	c.464C>T p.(Pro155Leu)		c.470C>T p.(Thr157Ile)	c.968T>G p.(Phe323Cys); c.1318 G>A p.(Glu440Lys)	c.980G>A p.(Arg327Gln) c.1255C>T p.(Arg419Trp)	c.1026G>C p.(Glu342Asp); c.774+5G>T		c.1036C>T p.(Arg346Cys); c.388-1G>C	c.1630C>T p.(Leu544Phe)	c.2051G>A p.(Arg684Gln); c.387+6T>C	c.387+6T>C; c.2140G>A p.(Ala714Thr)	c.1534G>A p.(Glu512Lys) c.773C>T p.(Ser258Leu)	c.1534G>A p.(Glu512Lys) c.773C>T p.(Ser258Leu)		c.464c>T p.(Pro155Leu)	c.773 C>T p.(Ser258Leu)	c.1010C>T p.(Ala337Val)	c.695+3A>G; c.968T>G p.(Phe323Cys)	c.695+3A>G; c.1285C>T p.(Arg429∗)	c.773C>T p.(Ser258Leu); c.1838C>T p.(Pro613Leu)	c.1274A>G p.(Glu425Gly)	c.1354C>T p.(Arg452Trp); c.1678G>C p.(Asp560His)	c.470G>C p.(Thr157Arg); c.2051C>T p.(Arg684Gln)	c.1679A>C p.(Asp560Ala); c.1036C >T p.(Arg346Cys)	c.470C > G p.(Thr157Arg); c.2143G > A p.(Glu715Lys)	c.845C>T p.(Pro282Leu); c.695+3A>G		
**Zigosity**	hom	hom		hom	c.het	c.het	c.het		c.het	hom	c.het	c.het	c.het	c.het		hom	hom	hom	c.het	c.het	c.het	hom	c.het	c.het	c.het	c.het	c.het		
**Age sex**	12m F	11y M	10y M	10m M	20y3m M	16m M	13y F	7y2m M	2y F	2y1m M	6y M	12y6m F	13y F	27y M	22y11m M	18m, M	4y11m	8y	6m, M	3.5m, F	10y, M	20y5m, M	10y3m, F	5y3m, F	2y6m, F	11m, M			
**Alive**	−	+	+	−	+	+	+	+	+	+	+	+	+	+	+	−	+	+	−	−	+	+	+	+	+	+	−	−	21/28 (75%)
**DD/ID**	Severe	Severe	Severe	Severe	Severe	Moderate	Moderate	Moderate	Severe	Normal till 1y	Severe	Severe	Moderate	Moderate	Moderate	Severe	Moderate (normal till 3y)	Severe	+	+	Moderate	Severe	Normal till 15m	Severe	Severe	+	+	+	28/28 (100%)
**Regression**	−	−	−	−	+	+	+	−	−	+	+	−	−	−	−	+	+	+	−	−	−	−	+	−	+	−	−	−	10/28 (36%)
**Non-ambulatory**	+	+	+	a	−	+	+ (post regression)	+	−	−	+	−	−	−	−	+	−	−	a	a	−	−	−	+	+	a	a	a	10/22 (45%)
**Non-verbal**	+	−	−	a	+	+	−	−	a	+	−	+	−	−	−	+	+	−	a	a	+	−	+ (normal till 15m)	+	+	a	a	a	11/21 (52%)
**Epilepsy**	+	+	+	+	−	−	−	−	−	+	+	−	−	−	−	+	−	+	+	+	+	−	+	−	+	+	−	−	14/28 (50%)
**Cerebellar abnormalities**	+	+	NA	+	+	+	+	NA	+	+	+	+	NA	NA	NA	NA	NA	−	−	+	+	+	+	−	−	+	−	−	
**BG signal alterations**	+	+	NA	+	+	+	−	NA	+	+	+	−	NA	NA	NA	NA	NA	+	−	−	+	+	−	+	−	+	+	−	
**Reduced WM volume with VE**	Moderate	Moderate	NA	Severe	−	−	−	NA	−	Mild	−	Moderate	NA	NA	NA	NA	NA	+	−	Moderate	−	−	−	−	+	−	−	−	
**CSF spaces Enlargement**	Mild	Mild	NA	Severe	−	−	−	NA	Mild	Mild	Mild	Mild	NA	NA	NA	NA	NA	Severe	Mild	Mild	−	−	−	−	−	Severe	−	−	
**Others**	CCH	PH	NA	CCH, midbrain atrophy, PH, brainstem signal alterations	−	MRS: small lactate peak	−	NA	−	CCH, midbrain atrophy	−	CCH, PH, Midbrain atrophy, IOND	NA	NA	NA	NA	NA	CCH	−	CCH, focal WM lesion MRS: lactate peak	−	Focal periventricular WM signal alterations	−	CCH	−	Brainstem signal alterations	CCH	−	
**OFC (SDS)**	−1.6	−3.9	−4.9	−6.1	+2.0	−0.2	+0.0	−1.0	−0.3	−3.3	−1.1	+3.3	+2.3	+2.3	+2.0	−2.8	−1.1	−3.0	−0.4	NA	−1.4	NA	−4.1	NA	−2.0	−2.8	NA	NA	8/23 (35%) microcephalic
**Axial Hypotonia**	+	+	+	+	+	+	+	+	+	+	+	+	+	−	−	+	NA	+	+	+	+	−	+	+	+	+	+	+	24/27 (89%)
**Limb hypertonia**	+	+	+	+	+	+	+	+	−	+	−	+	−	−	−	+	NA	−	−	+	+	−	+	+	−	+	+	+	18/27 (67%)
**Cerebellar deficits**	−	+	+	−	+	−	+	+	+	+	−	+	−	−	−	+	NA	−	−	+	−	−	−	+	+	NA	a	a	12/24 (50%)
**Dystonia**	−	+	+	+	+	+	−	−	−	+	+	+	−	−	−	+	NA	+	+	+	−	−	+	−	NA	NA	NA	NA	13/23 (56%)
**Cardiac hypertrophy**	+	−	−	+	−	−	−	−	−	−	−	−	−	−	−	−	NA	−	−	+ Cardiac arrest	−	−	−	NA	−	−	NA	NA	3/24 (12%)
**dRTA**	+	−	−	−	−	−	−	−	−	−	−	−	−	+	+	−	NA	+	−	−	−	+	−	−	+	−	NA	NA	6/25 (24%)
**Increased blood lactate**	+	+	+	+	NA	+	NA	NA	NA	+	+	−	NA	NA	NA	−	NA	+	+	+	+	−	+	+	+	+	+	+	17/20 (85%)
**Visual impairment**	+	−	−	−	−	−	+	+	−	−	+	−	+	−	−	−	NA	−	+	+	−	−	−	NA	NA	NA	NA	NA	7/22 (32%)
**SNHL**	−	−	−	−	−	−	−	−	−	−	−	−	−	−	−	−	−	NA	−	+	−	+	−	+	−	−	NA	NA	3/25 (12%)

*BG*, basal ganglia; *c.het*, compound heterozygous; *CCH*, corpus callosum hypoplasia; *CSS*, cerebral subarachnoid spaces; *DD*, developmental delay; *dRTA*, distal renal tubular acidosis; *F*, female; *hom*, homozygous; *GTC*, generalized tonic-clonic; *H*, hemispheres; *ID*, intellectual disability; *IOND*, inferior olivary nuclei degeneration; *m*, months; *M*, male; *MRS*, magnetic resonance spectroscopy; *NA*, not available; *PH*, pontine hypoplasia; *SNHL*, sensorineural hearing loss; *y*, years; *V*, vermis; *VE*, ventricular enlargement; *WM*, white matter.

a Too young for this developmental milestone/evaluation.

The identified variants are rare in human population variant databases (with allele frequency ranging from 0 to 0.000424 in 1,623,000 alleles across multiple databases) and have never been observed homozygously in healthy individuals (databases are listed in Supplemental Table 1). The majority of missense variants are predicted to have deleterious effects according to several predictive in silico tools. The splice variants c.774+5G>T and c.388-1G>C are predicted to affect transcript processing by in silico analysis (Human Splicing Finder, SpliceAI).^19^^,^^20^ Of note, a c.387+6T>C was found in the compound heterozygote state in 2 unrelated individuals of family 9 and 10. Complementary DNA studies using total RNA from peripheral blood cells of individuals II:1 of family 9 showed that the variant c.387+6T>C causes aberrant splicing with consequent deletion of exon 3 and 4 (Supplemental Figure 2). The American College of Medical Genetics and Genomics classification is reported in Supplemental Table 1. Specifically, we used the CADD, Polyphen-2 and Mutation Taster to apply the PP3 score. We also applied PP4 considering that all patients have been sequenced by highly sensitive methods and no other pathogenic or likely pathogenic variants were identified in genes known to be associated with neurological phenotypes.

### Clinical and neuroradiological characteristics of the affected individuals

This cohort consists of 18 affected individuals from 15 unrelated families, 6 of which were consanguineous (Table 1). All but 1 individual had moderate (*n* = 7) or severe (*n* = 10) developmental delay/intellectual disability. Psychomotor regression was noticed in 8 individuals, including 2 with early normal development. The regression of developmental milestones occurred within the first year of life for 3 individuals, typically triggered by intercurrent illnesses. Three individuals died within the first 2 years of life because of cardiorespiratory failure in the context of infection or palliative care. At the last evaluation, only 4 individuals were able to walk without support and 8 were nonverbal. Seven individuals had behavioral abnormalities, including 1 with a diagnosis of autism spectrum disorder. With the exception of 2 individuals, all others presented with muscular hypotonia since birth and 11 developed limb hypertonia with brisk reflexes. Nine individuals demonstrated cerebellar signs, including ataxia, tremor, titubation, dysmetria, and oculomotor apraxia. Ten patients had dystonia and other extrapyramidal movements. Six developed postnatal microcephaly, whereas 1 had macrocephaly in the context of familial macrocephaly.

Epilepsy, including tonic-clonic seizures, myoclonic seizures, and infantile spasms, occurred in 8 individuals and was successfully controlled with anti-seizure medications in half of them. Four individuals had distal renal tubular acidosis. Biventricular cardiac hypertrophy was found in 2 individuals, 1 of whom also developed pulmonary hypertension. Five individuals had visual system dysfunction, whereas none had hearing impairment. Mild to significant elevation of lactic acid was found in 70% of the individuals for whom it was tested (7/10). Mild and non-specific dysmorphic features were noticed in a majority of individuals (Figure 1D).

**Figure 2:**
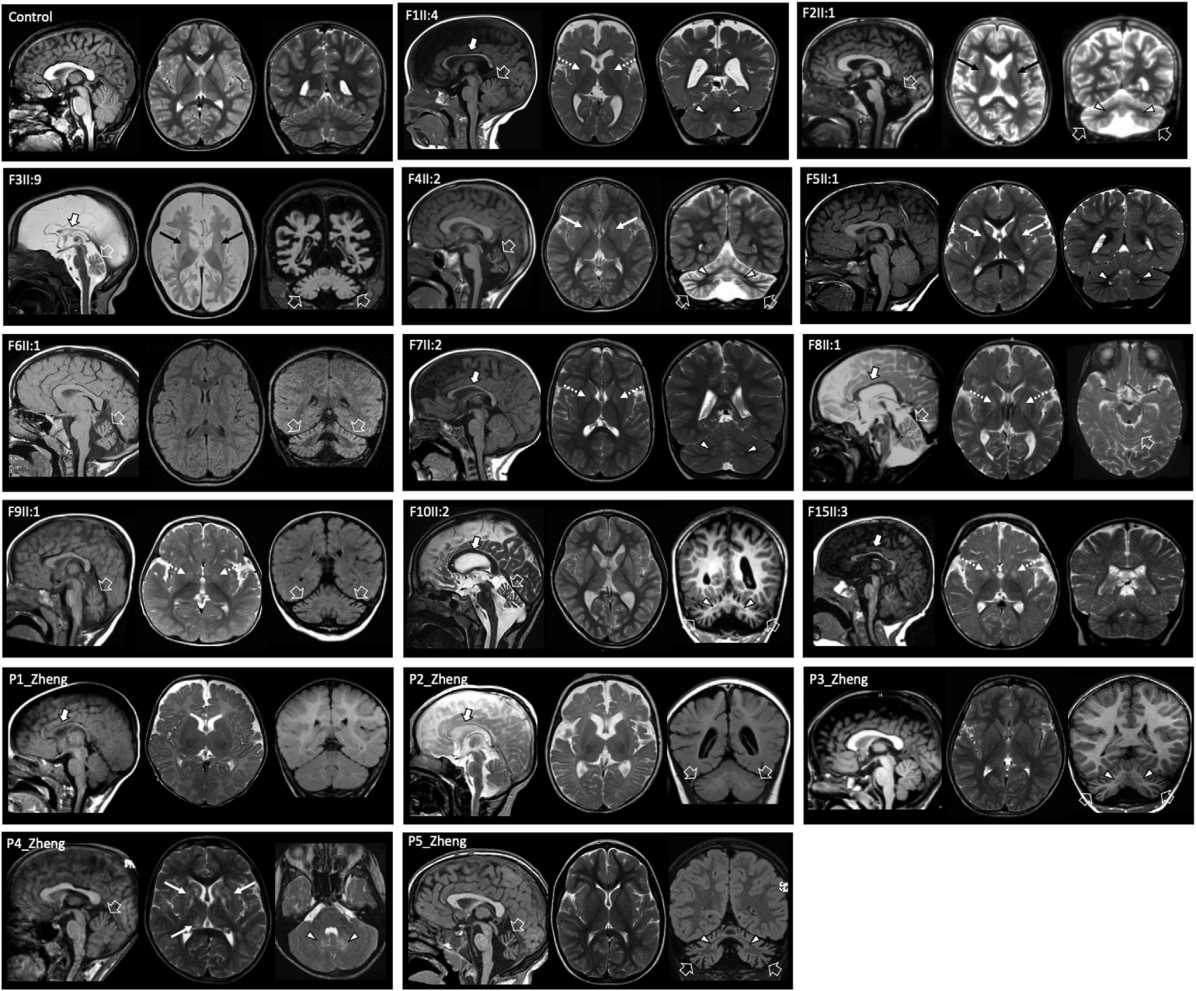
**Neuroradiological features of individuals with biallelic *TARS2* variants and of a control subject for comparison.** Most of the individuals present cerebellar atrophy (open arrows) variably associated with cerebellar cortex signal alterations and dentate nuclei T2/FLAIR hyperintensity (arrowheads). There is mild-to moderate white matter volume loss and/or enlargement of the cerebral CSF spaces in most individuals. The corpus callosum is thin in 8 individuals (thick arrows). Note the faint T2 signal abnormalities in the globi pallidi (dashed arrows) and putamen/caudate nuclei (thin arrows). CSF, cerebrospinal fluid.

Brain MRI was abnormal in all individuals for whom it was available (Figure 2), revealing mild to severe cerebellar atrophy with prevalent vermian involvement in 12 of 16 (75%) cases. Mild to moderate enlargement of the cerebral subarachnoid spaces, especially in the frontotemporal regions, was noted in 10 of 16 (62.5%) individuals. Cerebellar cortex T2/FLAIR hyperintensity and dentate nuclei signal alterations were present in 4 of 16 (25%) and 9 of 16 individuals (56.2%), respectively. Faint T2 signal alterations of the globi pallidi were detected in 8 of 16 (50%) individuals, whereas caudate nuclei/putaminal lesions were noted in 5 of 16 (31.3%). Reduced white matter volume with secondary enlargement of the lateral ventricles and thinning of the corpus callosum was present in 7 of 16 (43.7%) cases. In 1 individual (individual 2 of Zheng et al. 2022) a small lesion with restricted diffusion in the left frontal periventricular white matter was noted at presentation, at 1 month of age.^11^ Mild midbrain atrophy and pontine hypoplasia were noted in 3 of 16 (18.7%) individuals. Brainstem signal abnormalities were present in 2 of 16 (12.5%) cases. A spinal MRI from one individual was unremarkable. Magnetic resonance spectroscopy from 2 individuals showed lactate peak at the level of the basal ganglia in 1 of them. Only 2 patients underwent muscle biopsy showing features of complex I deficiency in 1 of them. Clinical features are summarized in Figure 1E and reported in more detail in Supplemental Table 2, also including updated data of the previously described cohorts.

### *TARS2* mutants F323C, R327Q, E342D, and R346C disrupt interaction of TARS2 with Rag GTPases

**Figure 3:**
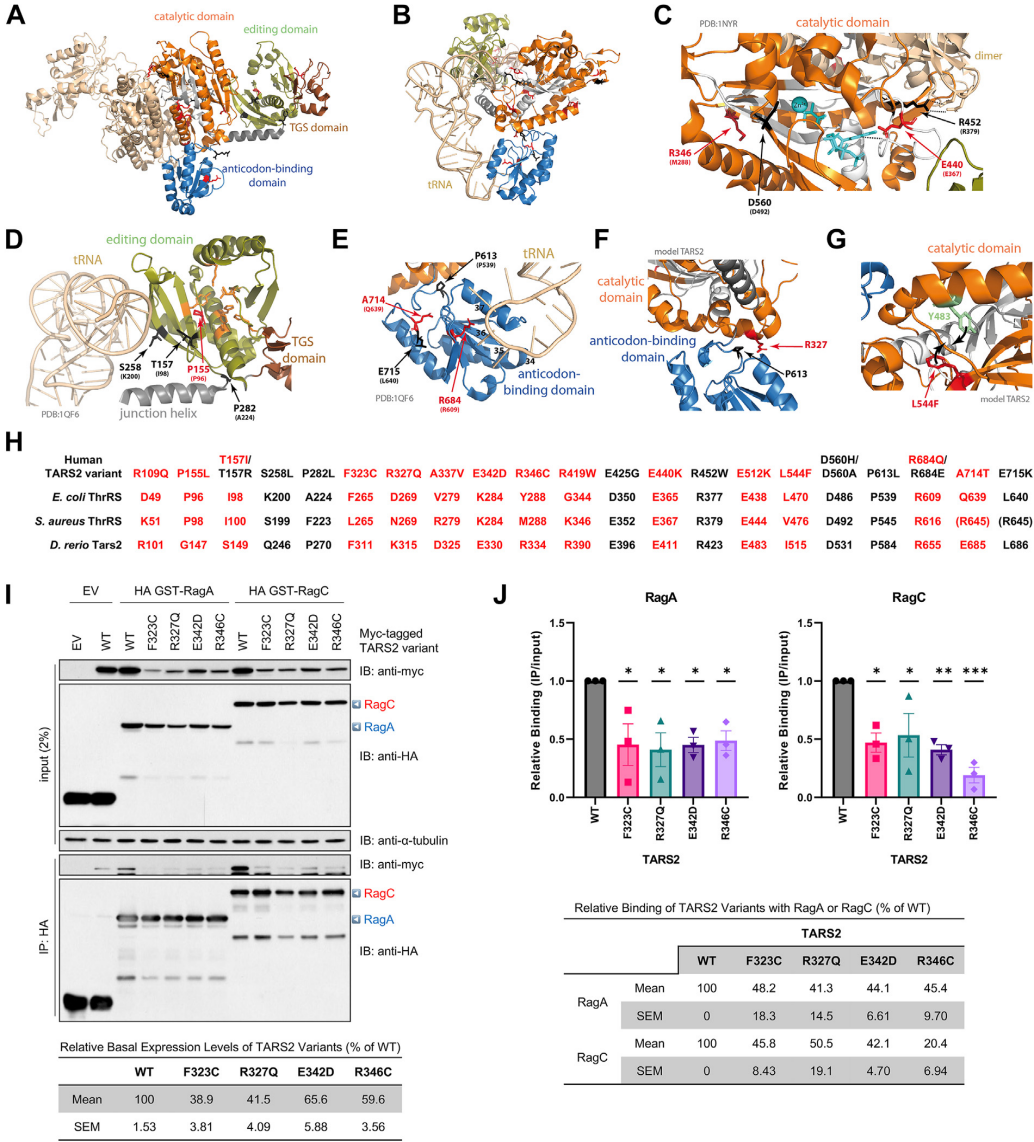
**Protein modeling and interaction of mutant *TARS2* with Rag GTPase.** A. Structure of the dimeric ThrRS from *Staphylococcus aureus* (PDB: 1NYR;^14^). Functional domains are named and colored in 1 monomer. B. Structure of one monomer of the *Escherichia coli* ThrRS, in complex with cognate tRNA with the CCA extremity of the tRNA pointing into the catalytic core (PDB: 1QF6;^15^). C-G. Structural environment of the wild-type residues and theoretical impact of their variants. Structural templates are indicated in light gray. The 3D model of human TARS2 is from misynpat.org.^16^^,^^18^ In all models, motifs 1, 2, and 3 that build the catalytic core are colored in white. Residues found mutated are shown in stick representation and colored in red for those reported in the present study and in black for those reported elsewhere (see Figure 1 and Supplemental Table 2 for references), with the corresponding amino acid from either *S. aureus* or *E. coli* indicated in brackets. C. Zoom into the catalytic core where catalytic Zn^2+^ and small substrates (aminoacyl-adenylate aa∼AMP and PPi) are shown in cyan. D. Zoom into the editing domain. Catalytic residues for editing, as identified in,^21^ are shown in orange. E. Zoom into the anticodon-binding domain. Key nucleotides from the tRNA anticodon loop are numbered (34-37). F. Zoom into the interface between the catalytic domain and the anticodon-binding domain. G. Example of possible local distortion engendered by the L544F variant. All molecular representations were prepared with PyMOL (Schrödinger, Inc.). H. Correspondence of residues of human (Uniprot: Q9BW92), *E. coli* (Uniprot: P0A8M3), *S. aureus* (Uniprot: Q2YTA7), and *D. rerio* (NCBI: XP_021322466.1) in the 3 structural templates as established using the multiple sequence alignment. I. Co-immunoprecipitation (co-IP) interaction of mutant *TARS2* variants with Rag GTPases. HEK293T cells were transfected with the indicated plasmids. Co-IP was performed using an anti-hemagglutinin (HA) antibody. Triangles on the right indicate the full-length RagA (blue) and RagC (red). α-tubulin is shown as a loading control. EV, empty vector; WT, wildtype. J. Mean ± SEM of relative binding of TARS2 with RagA (left) or RagC (right) from 3 independent co-IP experiments is shown. ∗*P* < .05; ∗∗*P* < .01; ∗∗∗*P* < .001 (one-way ANOVA followed by Dunnett’s post hoc test).

We performed detailed molecular modeling as described in Supplemental Results, Supplemental Figure 1, and shown in Figure 3A-H. It has previously been reported that TARS2 is required for the activation of mTORC1, a master regulator of cell growth and proliferation.^12^ mTORC1 is localized to and activated at the lysosomal surface in response to amino acid availability via Rag GTPases.^22^ Upon threonine supplementation, TARS2 preferentially binds RagC^GTP^ and promotes the conversion of inactive Rag GTPases (RagA^GDP^/RagC^GTP^) into the active form (RagA^GTP^/RagC^GDP^). This is sufficient to recruit mTORC1 to lysosomes.^12^ Notably, TARS2^301-381^, an intermediate region between the editing and catalytic core domains in the TARS2 protein, is crucial for interaction with RagC. Given that 4 *TARS2* variants, namely, F323C, R327Q, E342D, and R346C, occur within this region, we tested whether these amino acid changes interfere with the interaction between TARS2 and Rag GTPases. Despite being cloned from the same wild-type (WT) backbone, the TARS2 variants had reduced basal expression levels (ranging from 38.9 ± 3.81 [F323C] to 65.6 ± 5.88% [E342D] of WT; Figure 3I, input), probably because of intrinsic instability. Nonetheless, co-IP data showed that the relative binding of all 4 *TARS2* variants to RagA (ranging from 41.3 ± 14.5 [R327Q] to 48.2 ± 18.3% [F323C]; Figure 3J, upper left) or RagC (ranging from 20.4 ± 6.94 [R346C] to 50.5 ± 19.1% [R327Q]; Figure 3J, upper right) was significantly decreased compared with WT control. These results indicate that the *TARS2* variants impede interaction with Rag GTPases, presumably leading to subsequent suppression of amino acid-dependent mTORC1 activation.

### Functional studies in zebrafish

#### Functional analysis of TARS2 loss of function in zebrafish

**Figure 4:**
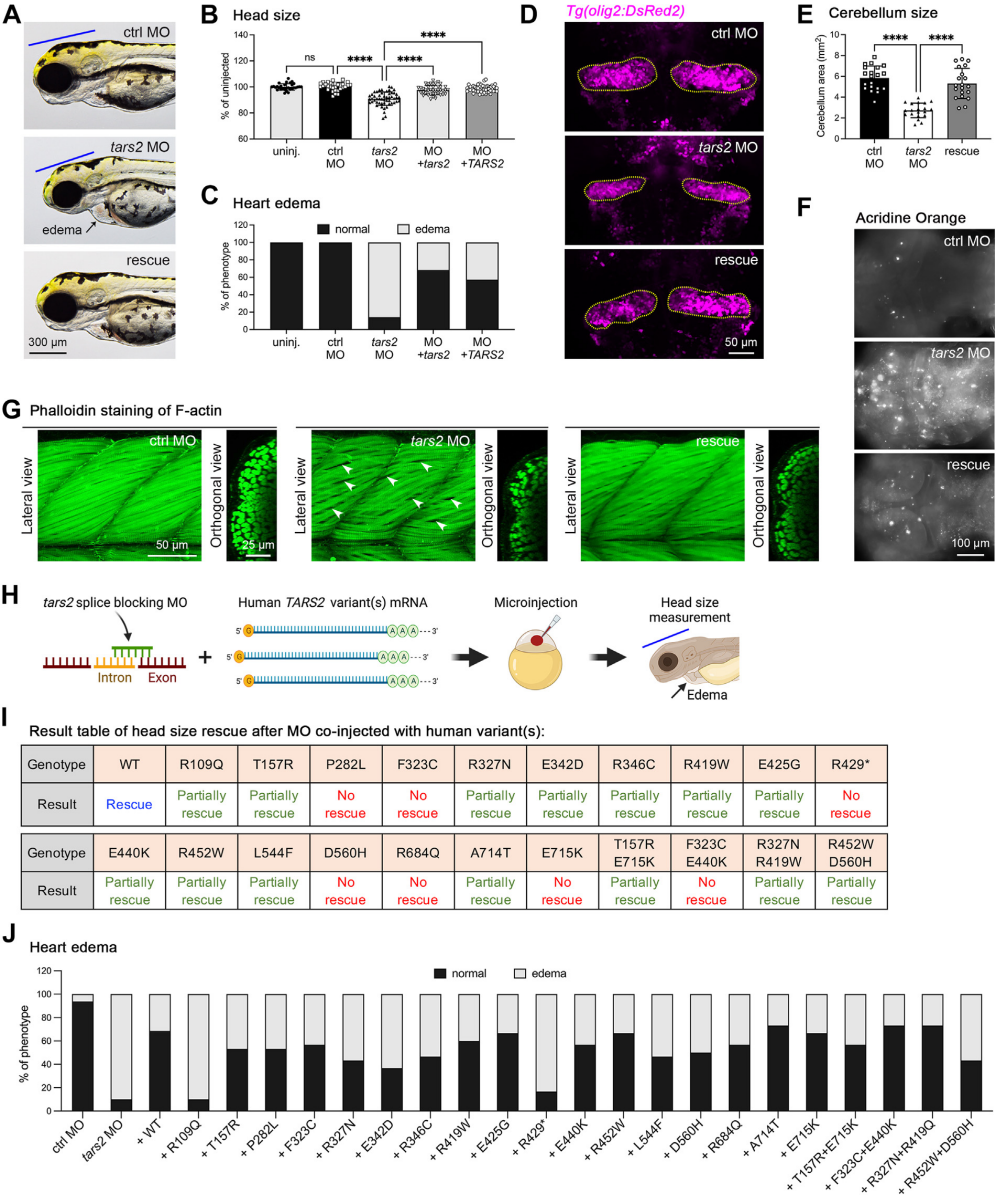
**Functional testing of *TARS2* variants in zebrafish.** A. Representative images of an embryo injected with control MO (ctrl MO), *tars2* MO, or *tars2* MO rescue at 3 dpf. Lateral view, anterior to the left. The blue line indicates brain size, and the black arrow indicates heart edema. B. Quantification of head size from uninjected (*n* = 30 animals), ctrl MO (*n* = 31 animals), *tars2* MO (*n* = 52 animals), *tars2* MO + zebrafish *tars2* RNA (+ *tars2*) (*n* = 41 animals), and *tars2* MO + human *TARS2* RNA (+ *TARS2*) (*n* = 52 animals) embryos at 3 dpf. Each symbol represents 1 animal. Values were calculated as percentage of the mean value of uninjected embryos. Error bars = mean ± SD. C. Quantification of heart edema. Animals were collected according to phenotype as shown in the image and calculated as the percentage of total animals. D. Representative cerebellum image of *Tg(olig2:DsRed)* of ctrl MO, *tars2* MO, and *tars2* MO rescue at 3 dpf. The red fluorescence color was replaced with pseudo-magenta color. E. Quantification of cerebellum area as indicated in (D). *n* = 10 animals. Error bars = mean ± SD. F. Acridine orange was used to stain brain in ctrl MO, *tars2* MO, and *tars2* MO rescue at 3 dpf. Scale bar = 100 μm. G. Confocal projections of phalloidin stained muscle fibers in the trunk region of ctrl MO, *tars2* MO, and *tars2* MO rescue. The anterior is shown to the left and dorsal at the top. The right panels show orthogonal views, dorsal to the top. H. Experimental approach for functionally characterizing human *TARS2* variants. In vitro synthetic human *TARS2* variant mRNA was mixed with zebrafish *tars2* splice-blocking MO and microinjected into 1-cell stage embryos, followed by phenotypic evaluation at 3 dpf. The blue line indicates head size and the black arrow indicates heart edema. I. Summary of rescue results with the mean value for each group (from Figure S6). No rescue means the mean value of the group is close to the mean of *tars2* MO group, and the statistic shows no significance compared with *tars2* MO group. Partially rescued means the mean value of the group is higher than the mean of *tars2* MO group but lower than the mean of uninjected control group, and the statistical difference shows significance compared with *tars2* MO but also shows significance compared with the uninjected control group. Rescue means the mean value of the group is close to the uninjected control and the statistical difference shows no significance compared with uninjected control group. J. Quantification of heart edema. Animal’s phenotypes were quantified as shown in the image and the percentage of total animals was calculated. In (B and E), one-way ANOVA with Tukey’s multiple comparison correction is shown: ns, not significant *P* ≥ .05, ∗*P* < .05, ∗∗*P* < .01, ∗∗∗*P* < .001 and ∗∗∗∗*P* < .0001 compared with *tars2* MO group.

The *tars2* mRNA in zebrafish is maternally distributed and continually expressed throughout embryogenesis (Supplemental Figure 3). To examine the in vivo function of Tars2 and its pathophysiology in individuals, we designed a *tars2* splice-blocking morpholino (MO) to block precursor mRNA processing (Supplemental Figure 4A). The efficacy of the MO knockdown was confirmed through reverse transcription–polymerase chain reaction, revealing a decrease in the WT *tars2* transcript compared with uninjected and control MO embryos (Figure S4B). Knockdown of *tars2* in zebrafish embryos (morphants) via MO led to a significant reduction in head size (Figure 4A and B) and heart edema (Figure 4C) compared with uninjected and control MO embryos, suggesting that Tars2 is crucial for brain and heart development in zebrafish. To ensure phenotype specificity, we performed mRNA rescue, mitigating the morphant phenotypes by co-injecting synthetic WT zebrafish *tars2* or human *TARS2* mRNA along with MO; this restored the overall phenotypes (Figure 4A bottom panel), as well as normal head size and heart edema (Figure 4B and C). This evidence confirms that the observed phenotypes resulted from *tars2* downregulation and demonstrates functional conservation across species. Consequently, human *TARS2* mRNA was utilized in subsequent rescue experiments (labeled as a rescue in the Figures).

Given the cerebellar atrophy observed in many affected individuals, we used the transgenic reporter line, *Tg(olig2:DsRed2*), to examine cerebellum morphology. This line expresses the Olig2 transcription factor, crucial for developing primary motor neurons and oligodendrocytes. In our study, we observed a smaller cerebellum (marked by olig2+ cells) in *tars2* morphants compared with control morphants (see Figure 4D and E). Because TARS2 is known to regulate cell proliferation,^12^ and based on our previous research on KARS1,^23^ we speculated that the reduced head size in *tars2* morphants might be due to brain cell apoptosis. We conducted live cell brain imaging using acridine orange dye^24^ to investigate this and observed extensive apoptosis in *tars2* morphants (Figure 4F). Quantitative reverse transcription polymerase chain reaction analysis of apoptosis-related genes revealed significant upregulation of *gadd45aa* and *caspase-8* but not *tp53* and *caspase-9* (Figure S5A), indicating cell growth suppression and activation of extrinsic cell apoptosis pathways.^25^

Our study confirmed that zebrafish with reduced TARS2 function (*tars2* morphants) showed decreased myelination and increased seizure activity, as indicated by reduced *mbpa* and elevated *c-fos* expressions (Supplemental Figure 5B and C), respectively. This aligns with prior findings linking mitochondrial aaRS dysfunction to myelination and seizure phenotypes.^4^ We also discovered changes in mTORC1 signaling pathway in these morphants, with decreased *PGC1α* and increased *PPARα* levels (Supplemental Figure 5D and E), both regulated by mTORC1.^11^^,^^26^^,^^27^ However, unlike TARS1 reduction, TARS2 inhibition did not activate the expression of the unfolded protein response target, *atf4a* and *atf4b*^28^(Supplemental Figure 5F). Individuals with *TARS2* variants were found to have muscular hypotonia and movement disorders. Indeed, our WISH data revealed *tars2* mRNA expression in trunk muscles (Supplemental Figure 3E). In *tars2* morphants, the phalloidin staining of F-actin was diminished in the myotomes, indicating less condensed muscle fibers than control MO-injected morphants (Figure 4G), suggesting a muscular dysfunction that may result in the absence of coordinated locomotion. The observed morphological defects specific to the brain, eyes, heart, and trunk muscles in *tars2* morphants directly correlate with the *tars2* mRNA expression patterns during embryonic development. This further confirms that the tissue-specific phenotypes arise from a loss of Tars2 function.

#### Functional validation of TARS2 variants in zebrafish model

Next, we aimed to investigate the impact of previously identified variants on protein functionality in addition to those present in our cohorts. To test these variants, we created patient-specific mutations in human complementary DNA, and used these constructs as a template to synthesize mRNA using in vitro transcription. We assessed the effect of variants utilizing an mRNA rescue technique. In this method, we co-injected either WT or mutant mRNA with either control MO or *tars2* MO and then scored the brain and heart development phenotypes (Figure 4H). We employed a splice-blocking *tars2* MO, ensuring that it would not degrade the exogenous mRNA encoding human TARS2 utilized for rescue. We individually tested 17 variants. Because some variants appeared in a compound heterozygous state, we co-expressed mRNA encoding both variants. The head size of the animals rescued with variant mRNA was then compared with that of animals from the *tars2* morphant and *TARS2* WT rescue groups (Supplemental Figure 6). Six variants identified in humans in a homozygous state and 2 in a compound heterozygous state failed to rescue the head size phenotype. Conversely, 11 variants only partially rescued the phenotypes, suggesting that each variant is deleterious to protein function (Supplemental Figures 6 and 4I). Interestingly, we observed the most severe cardiac edema in the R109Q-rescued morphant, which is consistent with the discovery of significant biventricular hypertrophy in the individual carrying the c.326G>A p.(Arg109Gln) variant (II:IV of family 1, Figure 4J). Similarly, the loss-of-function variant p.(Arg429∗) could not rescue the cardiac phenotype. All other tested variants only partially mitigated the heart edema, with some degree of variation. To sum up, the pathogenicity of the 25 variants was confirmed through functional assays in a zebrafish model.

## Discussion

We report here 18 new individuals with biallelic variants in *TARS2* and review neuroimaging of 5 previously reported individuals,^11^ contributing to our understanding of TARS2-related mitochondrial disease. Symptoms include developmental delay, intellectual disability, regression, and brain atrophy, along with changes in basal ganglia signals and mitochondrial characteristics, such as lactic acidosis, cardiomyopathy, and distal renal tubulopathy. Clinically, these patients display hypotonia, cerebellar signs, varying degrees of spasticity, dystonia, seizures, and secondary microcephaly.

Previous studies classified the central nervous system (CNS) aaRSs disorders based on the main neurological phenotypes, suggesting that *TARS2* variants were mainly associated with a fatal infantile NDD.^9^ However, others have categorized ARS disorders based on the prevalent organ, subdividing disorders as follows: (1) affecting the CNS and other systems (2) and preferentially or exclusively other systems than the CNS (3 or 4).^7^ TARS2-related mitochondrial disease was placed in the first group and considered a prevalent epileptic encephalopathy disorder.

The present study and other recent reports reveal that the clinical phenotypes relating to biallelic *TARS2* variants are broader, with variable survival of individuals, occurrence of regression (*n* = 10/28, 36%), seizure (*n* = 14/28, 50%), secondary microcephaly (8/23, 34%), axial hypotonia (24/27, 89%), limb hypertonia (18/27, 67%), cerebellar deficits (12/24, 50%), and extrapyramidal movements (13/23, 56%). Notably, increased blood lactate is a consistent finding in all affected individuals for whom it was checked (17/20 85%), whereas cardiomyopathy and renal tubular acidosis have been observed only in 3 (12%) and 6 (24%) individuals, respectively. Of note, cardiomyopathy was previously reported only in 1 patient.^11^ Although 3 patients were previously reported with neurosensorial hearing loss, none of our cohort had hearing impairment.^11^^,^^18^ It is possible that these features may be underestimated, considering that no affected individual had specific work-up or that they may develop hearing impairment and cardiac and rental tubular dysfunction later. A cardiological evaluation, urine analysis for tubulopathy and hearing screening is warranted in patients with biallelic *TARS2* variants. One of the 2 patients who underwent muscle biopsy showed features of complex I deficiency similar to 4 previously reported patients who had multiple complex deficiency^9^^,^^11^ (Supplemental Table 2).

The most common neuroradiological finding in TARS2-related disorder was cerebellar atrophy in 75% of individuals and often associated with progressive frontotemporal cerebral atrophy. Notably, the cerebellum was initially normal in 1 infant who underwent brain MRI in the first months of life. Therefore, we hypothesize that cerebellar atrophy might develop later in TARS2-related disorders and be missed without follow-up MRI studies, as in the cases reported previously.^9^^,^^10^ Other frequent neuroimaging features in individuals with *TARS2* variants were signal alterations of the basal ganglia and loss of periventricular white matter volume, present in 68.5% and 43.7% of cases, respectively. In particular, approximately half of individuals presented signal alterations of the globi pallidi, whereas caudate nuclei and putaminal lesions were noted in one third of cases. In 1 individual, follow-up MRI at 16 months of age revealed progression of the basal ganglia lesions with associated lactate peaks on magnetic resonance spectroscopy and new dentate signal alterations characterized by restricted diffusion. A similar MRI pattern was also present in the individual described by Li et al,^10^ thus suggesting that *TARS2* variants might also be associated with a Leigh-like neuroimaging pattern, in keeping with the phenotypic heterogeneity of ARS2-related disorders.^7^^,^^8^

Several ARS2-related encephalopathies, including *CARS2*, *FARS2*, *MARS2*, *VARS2*, and *WARS2*, display similar neuroimaging features, indicating mitochondrial impairment.^29^Additionally, specific neuroimaging patterns in ARS2-related encephalopathies include peculiar leukoencephalopathies, such as those described in *DARS2-* and *EARS2-*related disorders^30^ or pontocerebellar hypoplasia reported in individuals with biallelic variants in *RARS2*.^31^ These features have not been identified in individuals with *TARS2* variants, and they could be valuable for differential diagnosis of these rare conditions.

It is noteworthy that 17 of the 24 families reported to date harbor recurrent *TARS2* variants and 5 of the current cohort have been previously reported (Figure 1A and B). There is a predominance of missense variants and only 1 patient previously reported^11^ was harboring 1 stop-gain in combination with a splicing variant, suggesting that total loss of TARS2 is likely lethal in humans. Some missense variants cluster, such as those in the TARS2-Rag GTPase binding region and in the editing and AB domains. Also, 2 splice variants recur in the cohort (c.387+6T>C, c.695+3A>G). Although we did not recognize a specific genotype-phenotype correlation, some interesting findings emerged. Two unrelated individuals harbor the same c.464C>T p.(Pro155Leu) variant that is associated with a severe phenotype, including intellectual disability, microcephaly, cerebellar dysfunction, and epilepsy. Interestingly, the c.1534G>A p.(Glu512Lys) and c.773C>T p.(Ser258Leu) variants were found in a compound heterozygous state in 3 individuals of 2 unrelated families (11 and 12), and the c.773C>T p.(Ser258Leu) was also found in homozygous state in family 14. These individuals had moderated intellectual disability and less severe motor and cerebellar dysfunction. Furthermore, all but 1 individual harboring compound heterozygous variants in the TARS2-Rag GTPase binding region showed basal ganglia and cerebellar signal alterations without cerebellar atrophy. Because most identified variants were compound heterozygous with either a different type of variants or missense variants affecting different domains, this challenges a possible genotype-phenotype correlation. Overall, our findings are in line with the wide phenotypic variability reported for most ARS2-related disorders, for which no genotype-phenotype correlations have been recognized. Previous studies have shown that some *TARS2* variants can affect amino acid activation, tRNA charging, and dimer formation.^11^^,^^32^ However, different results were noted, suggesting distinct impacts on protein stability and function depending on yet unknown related mechanisms.^11^ Structural analysis of the missense *TARS2* variants further highlights that, though some may have an obvious impact on the canonical aminoacylation function, for instance, p.(Glu440Lys) and p.(Arg684Gln), other variants are unlikely to disrupt this. Three of the variants lying in the TARS2^301-381^ region (Arg327Gln, Glu342Asp, and Arg346Cys) affect residues only conserved in metazoans (F323 is conserved more widely in the eukaryotes), suggesting involvement in a metazoan-specific function, such as regulation of mTORC1 signaling. TARS2 was recently shown to regulate the activation of mTORC1 signaling, controlling cell proliferation and mRNA translation in a threonine-dependent manner.^12^ We found that all 4 variants within the TARS2^301-381^ region had decreased binding to Rag GTPases, likely impairing mTORC1 activity. This might have a detrimental impact on several biological functions, including mitochondrial homeostasis, because mTORC1 controls the mitochondrial activity by selectively promoting translation of nucleus-encoded mitochondria-related mRNAs.^33^ Altogether, these results suggest a distinct pathomechanism related to dysfunction of the mTORC1 signaling pathway, at least for variants within the Rag GTPase binding domain.

Our detailed zebrafish study shows that zebrafish larvae with *TARS2*-related pathogenic variants display hallmark symptoms, such as cerebellar hypoplasia, reduced head size, heart edema, and diminished F-actin expression in muscle segments. This highlights the TARS2 deficiency-induced developmental issues in the CNS, heart, and musculoskeletal systems. We also observed reduced myelin-binding protein *mbpa* expression and increased seizure-specific *c-fos* levels, consistent with the common white matter changes and increased seizure susceptibility in most affected individuals.

Considering the pivotal role of TARS2 in regulating mTORC1 activity and our in vitro results, we aimed to assess the expression of downstream targets of mTORC1 signaling in vivo. As expected, we found that *PGC1α* and *PPARα* were downregulated and upregulated, respectively, in *tars2* knock-down animals, likely leading to dysregulation of mTORC1 signaling.

Most tested variants in the in vivo zebrafish model failed to phenotypically rescue, confirming that the identified human *TARS2* variants likely affect protein function. Taken together, our results suggest that TARS2 plays an essential role in early CNS development, possibly affecting neuronal proliferation and apoptosis through aberrant mTORC1 signaling.^34^ Consequently, these observations raise the question whether mTORC1 drug modulators could be therapeutic for individuals with TARS2-related disorder in the future.

In conclusion, we further delineate the molecular and clinical spectrum of the rare TARS2-related NDD. Improved understanding of the underlying mechanisms leading to these features will be crucial to develop future therapeutic strategies that prevent metabolic decompensations and comorbidities in affected individuals.

## Data Availability

Human variant data included in this study have been deposited in the ClinVar database.

## Conflict of Interest

Amber Begtrup, Hong Cui, and Aida Telegrafi are employees of GeneDx, LLC. All other authors declare no competing interests.

## Additional Information

Supplementary Table S3

TARS2 Variant Submission validated

## References

[1] Bonnefond L., Fender A., Rudinger-Thirion J., Giegé R., Florentz C., Sissler M. (2005). Toward the full set of human mitochondrial aminoacyl-tRNA synthetases: characterization of AspRS and TyrRS. Biochemistry.

[2] Figuccia S., Degiorgi A., Ceccatelli Berti C., Baruffini E., Dallabona C., Goffrini P. (2021). Mitochondrial aminoacyl-tRNA synthetase and disease: the yeast contribution for functional analysis of novel variants. Int J Mol Sci.

[3] Boczonadi V., Jennings M.J., Horvath R. (2018). The role of tRNA synthetases in neurological and neuromuscular disorders. FEBS Lett.

[4] Fine A.S., Nemeth C.L., Kaufman M.L., Fatemi A. (2019). Mitochondrial aminoacyl-tRNA synthetase disorders: an emerging group of developmental disorders of myelination. J Neurodev Disord.

[5] Sissler M., González-Serrano L.E., Westhof E. (2017). Recent advances in mitochondrial aminoacyl-tRNA synthetases and disease. Trends Mol Med.

[6] Orellana E.A., Siegal E., Gregory R.I. (2022). tRNA dysregulation and disease. Nat Rev Genet.

[7] González-Serrano L.E., Chihade J.W., Sissler M. (2019). When a common biological role does not imply common disease outcomes: disparate pathology linked to human mitochondrial aminoacyl-tRNA synthetases. J Biol Chem.

[8] Ognjenović J., Simonović M. (2018). Human aminoacyl-tRNA synthetases in diseases of the nervous system. RNA Biol.

[9] Diodato D., Melchionda L., Haack T.B. (2014). VARS2 and TARS2 mutations in patients with mitochondrial encephalomyopathies. Hum Mutat.

[10] Li X., Peng B., Hou C. (2020). Novel compound heterozygous TARS2 variants in a Chinese family with mitochondrial encephalomyopathy: a case report. BMC Med Genet.

[11] Zheng W.Q., Pedersen S.V., Thompson K. (2022). Elucidating the molecular mechanisms associated with TARS2-related mitochondrial disease. Hum Mol Genet.

[12] Kim S.H., Choi J.H., Wang P. (2021). Mitochondrial threonyl-tRNA synthetase TARS2 is required for threonine-sensitive mTORC1 activation. Mol Cell.

[13] Richards S., Aziz N., Bale S. (2015). Standards and guidelines for the interpretation of sequence variants: a joint consensus recommendation of the American College of Medical Genetics and Genomics and the Association for Molecular Pathology. Genet Med.

[14] Torres-Larios A., Sankaranarayanan R., Rees B., Dock-Bregeon A.C., Moras D. (2003). Conformational movements and cooperativity upon amino acid, ATP and tRNA binding in threonyl-tRNA synthetase. J Mol Biol.

[15] Sankaranarayanan R., Dock-Bregeon A.C., Romby P. (1999). The structure of threonyl-tRNA synthetase-tRNA(Thr) complex enlightens its repressor activity and reveals an essential zinc ion in the active site. Cell.

[16] Moulinier L., Ripp R., Castillo G., Poch O., Sissler M. (2017). MiSynPat: an integrated knowledge base linking clinical, genetic, and structural data for disease-causing mutations in human mitochondrial aminoacyl-tRNA synthetases. Hum Mutat.

[17] Gao X., Xin G., Tu Y. (2022). TARS2 variants causes combination oxidative phosphorylation deficiency-21: a case report and literature review. Neuropediatrics.

[18] He P., Wang Q., Hong X., Yuan H. (2023). Novel TARS2 variant identified in a Chinese patient with mitochondrial encephalomyopathy and a systematic review. Am J Med Genet A.

[19] Desmet F.O., Hamroun D., Lalande M., Collod-Béroud G., Claustres M., Béroud C. (2009). Human Splicing Finder: an online bioinformatics tool to predict splicing signals. Nucleic Acids Res.

[20] Jaganathan K., Kyriazopoulou Panagiotopoulou S., McRae J.F. (2019). Predicting splicing from primary sequence with deep learning. Cell.

[21] Dock-Bregeon A., Sankaranarayanan R., Romby P. (2000). Transfer RNA-mediated editing in threonyl-tRNA synthetase. The class II solution to the double discrimination problem. Cell.

[22] Dibble C.C., Cantley L.C. (2015). Regulation of mTORC1 by PI3K signaling. Trends Cell Biol.

[23] Lin S.J., Vona B., Barbalho P.G. (2021). Biallelic variants in KARS1 are associated with neurodevelopmental disorders and hearing loss recapitulated by the knockout zebrafish. Genet Med.

[24] Tucker B., Lardelli M. (2007). A rapid apoptosis assay measuring relative acridine orange fluorescence in zebrafish embryos. Zebrafish.

[25] Jin S., Tong T., Fan W. (2002). GADD45-induced cell cycle G2-M arrest associates with altered subcellular distribution of cyclin B1 and is independent of p38 kinase activity. Oncogene.

[26] SenGupta S., Peterson T.R., Laplante M., Oh S., Sabatini D.M. (2010). mTORC1 controls fasting-induced ketogenesis and its modulation by ageing. Nature.

[27] Cunningham J.T., Rodgers J.T., Arlow D.H., Vazquez F., Mootha V.K., Puigserver P. (2007). mTOR controls mitochondrial oxidative function through a YY1-PGC-1alpha transcriptional complex. Nature.

[28] Castranova D., Davis A.E., Lo B.D. (2016). Aminoacyl-transfer RNA synthetase deficiency promotes angiogenesis via the unfolded protein response pathway. Arterioscler Thromb Vasc Biol.

[29] Roux C.J., Barcia G., Schiff M. (2021). Phenotypic diversity of brain MRI patterns in mitochondrial aminoacyl-tRNA synthetase mutations. Mol Genet Metab.

[30] Al Balushi A., Matviychuk D., Jobling R., Salomons G.S., Blaser S., Mercimek-Andrews S. (2020). Phenotypes and genotypes of mitochondrial aminoacyl-tRNA synthetase deficiencies from a single neurometabolic clinic. JIMD Rep.

[31] Nuovo S., Micalizzi A., Romaniello R. (2022). Refining the mutational spectrum and gene-phenotype correlates in pontocerebellar hypoplasia: results of a multicentric study. J Med Genet.

[32] Wang Y., Zhou X.L., Ruan Z.R., Liu R.J., Eriani G., Wang E.D. (2016). A human disease-causing point mutation in mitochondrial threonyl-tRNA synthetase induces both structural and functional defects. J Biol Chem.

[33] Morita M., Gravel S.P., Chénard V. (2013). mTORC1 controls mitochondrial activity and biogenesis through 4E-BP-dependent translational regulation. Cell Metab.

[34] Chan W.Y., Lorke D.E., Tiu S.C., Yew D.T. (2002). Proliferation and apoptosis in the developing human neocortex. Anat Rec.

