# Supplementary material for: Clinical, neuroradiological, and molecular characterization of mitochondrial threonyl-tRNA-synthetase (TARS2)-related disorder
Source: Genet Med. 2023 Nov;25(11):100938. doi: 10.1016/j.gim.2023.100938 (PMC11157694; doi:10.1016/j.gim.2023.100938)

Supplementary Information

Supplementary methods 1
Conservation analysis of the missense variants 1
Cell culture, transient transfection, and co-immunoprecipitation 2
Preparation of mutant TARS2 plasmids, cell culture, transient transfection, and co-immunoprecipitation 2
Zebrafish functional studies 2
Whole-mount in situ hybridization (WISH) 3
Morpholino-mediated knockdown of *tars2* expression 3
Cloning, site directed mutagenesis, synthesis of capped mRNA and microinjections 3
Morphological phenotyping and imaging 4
Immunohistochemistry 4
RNA extraction, reverse transcription and quantitative PCR (RT-qPCR) 4
Statistical analysis 5
Supplementary Results 5
Analysis of amino-acid conservation of TARS2 residues impacted by disease-associated missense variations 5
Analysis of the structural environment of the residues affected by missense variants 6
***Zebrafish tars2* mRNA was strongly expressed in developing brain** 7
Supplementary Tables and Figures 8
Supplementary References 10

Supplementary methods

Conservation analysis of the missense variants

TARS protein sequences from 50 organisms, which are representative of bacterial, archaeal and eukaryotic (cytosolic and mitochondrial) phylogenic diversity, are aligned using the MAFFT program[1]. The multiple sequence alignment (MSA) is generated inside the workbench ORDALIE (ORDered ALIgnment Information Explorer; to be published (Moulinier et al., 2021) and is drawn up using SnapGene® Viewer.

Cell culture, transient transfection, and co-immunoprecipitation

HEK293T cells were maintained in Dulbecco's Modified Eagle Medium (DMEM) (Wisent) supplemented with 10% fetal bovine serum (FBS) (Wisent) and 1% streptomycin-penicillin (Wisent) in a humidified atmosphere containing 5% CO2 at 37℃. Transient transfection of plasmids was performed using Lipofectamine 2000 (Invitrogen) according to the manufacturer's instructions. For co-IP assay, 24 h after transfection, cells were washed by ice-cold PBS and collected by scraping in the pre-chilled lysis buffer (40 mM HEPES-KOH pH 7.5, 120 mM NaCl, 1 mM EDTA, 50 mM NaF, 0.3% CHAPS, supplemented with a complete EDTA-free protease inhibitor tablet and phosphatase inhibitor cocktail). Cells were lysed using a rotator for 20 min at 4℃, and the lysates were incubated with Protein G agarose beads (Millipore) for at least 30 min at 4℃. Pre-cleared lysates were incubated with anti-hemagglutinin (HA).11 epitope tag antibody (Covance) for 1 hour at 4℃, followed by incubated with Protein G agarose beads overnight at 4℃. Beads were washed with wash buffer (lysis buffer supplemented with 50 mM HEPES-KOH pH 7.5, 150 mM NaCl) three times for 10 min each. Proteins bound to beads were eluted in 2x SDS sample buffer. TrueBlot anti-rabbit IgG HRP and anti-mouse IgG HRP (Rockland) were used as secondary antibodies for immunoblotting to eliminate signals from IgG heavy and light chains.

Preparation of mutant TARS2 plasmids, cell culture, transient transfection, and co-immunoprecipitation

Plasmids overexpressing the F323C, R327Q, E342D, and R346C *TARS2* mutants were derived from pcDNA4 *TARS2*-His/Myc wild-type (WT) plasmid[13] using the QuikChange Lightning Site-Directed Mutagenesis Kit (Agilent Inc. USA) according to the manufacturer's instructions. Cell culture, transient transfection, and co-immunoprecipitation (co-IP) assay were performed as described previously[13] with minor modifications as described in supplementary methods.

Zebrafish functional studies

We performed zebrafish experiments in WT strain NHGRI-1 according to the protocol approved by the Institutional Animal Care Committee (IACUC) of Oklahoma Medical

Research Foundation (22-76). All animals were raised and maintained in an Association for Assessment and Accreditation of Laboratory Animal Care (AAALAC) accredited facility under standard conditions.

Whole-mount in situ hybridization (WISH)

The whole-mount in situ hybridization was performed using a method described earlier[2]. Briefly, 1100-bp *tars2* sequence was PCR amplified from zebrafish cDNA using primers pairs listed in Table S3. Amplified DNA fragment was used as a template to synthesize digoxigenin-UTP-labeled antisense riboprobe. 24 hours post-fertilization (hpf) stage wild-type embryos were incubated with 0.003% 1-Phenyl-2-thiourea (PTU) (Millipore Sigma, MO, USA) to prevent pigments formation. Embryos were then fixed with 4% (V/V) paraformaldehyde (PFA)/ phosphate-buffered saline (PBS), followed by dehydration using increasing methanol series, then stored in 100% methanol at -20°C for at least overnight. Next day, samples were rehydrated with decreasing methanol series and permeabilized by 10 µg/mL proteinase K as described earlier[3]. The color development was performed using the BM purple alkaline phosphatase substrate (Millipore Sigma, MO, USA).

Morpholino-mediated knockdown of *tars2* expression

The zebrafish *tars2* antisense splice-blocking morpholino oligonucleotides (*tars2* MO, 5'-TGCAAACGTACCCAGAGTGTGTACC-3') and the standard control MO (ctrl MO, 5'-CCTCTTACCTCAGTTACAATTTATA-3') were synthesized by GeneTools Inc. (Philomath, OR, USA). Approximately 8 nanogram of MO oligonucleotides were injected into 1-cell stage embryos as per guidelines for morpholino use in zebrafish[4].

Cloning, site directed mutagenesis, synthesis of capped mRNA and microinjections

The full-length coding sequence of zebrafish *tars2* was amplified from WT zebrafish cDNA. Full-length human *TARS2* was PCR amplified from cDNA clone (# MHS6278-202827455, Horizon Discovery Ltd.) using Q5 high-fidelity DNA polymerase (New England Biolabs, Ipswich, MA, USA). The purified PCR products were cloned into the

pCS2+ plasmid (a kind gift by Dr. David Turner lab, University of Michigan). Variants from affected individual were introduced in zebrafish *tars2* cloned into pCS2+ at homologous positions using Quick Change XL site directed mutagenesis kit (Agilent Inc.), and primers listed in table S3. Wild-type *tars2* and *tars2* variant-specific plasmids were verified by sequencing, linearized, and then used as templates for synthesizing capped messenger RNA (mRNA) as described earlier [3]. One hundred fifty picogram of wild-type mRNA or variant-specific mRNA, together with *tars2* MO was injected for the rescue experiments. The amount of mRNA in a mixture was adjusted to achieve the lowest number of dysmorphic embryos and highest efficiency.

Morphological phenotyping and imaging

Morphological phenotyping was carried out by randomly selecting animals from control MO, *tars2* MO and *tars2* MO rescue groups at 3 dpf and anesthetized in Tricaine/MS-222 (Sigma-Aldrich, MO, USA). Animals were oriented within an agarose cavity mold or 2% methylcellulose (Sigma Aldrich, MO, USA) under a stereomicroscope and imaged using either an Olympus SZX12 stereomicroscope fitted with an Olympus DP71 color digital camera (Olympus, Tokyo, Japan) or a high-definition camera Nikon DS-Fi2 mounted on a Nikon SMZ18 (Nikon, Japan) stereomicroscope. The head and eye sizes were measured from scale-calibrated images using ImageJ software (NIH).

Immunohistochemistry

Phalloidin staining was performed as described earlier [3]. Whole-mount embryo cell apoptosis was visualized using acridine orange (AO) as per methods described previously [5]. Briefly, 20 animals for each group at 72 hpf were incubated in 10 μg/mL AO (Invitrogen, Eugene, OR, USA) in E3 buffer for 1 hour, and then rinsed thoroughly in E3 buffer for three times. Next, animals were anaesthetized and mounted in 1.5% low melting point agarose and images were acquired with a Zeiss LSM-710 confocal microscope.

RNA extraction, reverse transcription and quantitative PCR (RT-qPCR)

Total RNA was extracted using the TRIzol Reagent (Thermo Fisher Scientific, CA, USA) and purified using RNA clean and concentrator-5 kit (Zymo Research, CA, USA) as per

manufacturer's instructions. The cDNA was synthesized using iScript RT Supermix (Bio-Rad, USA), and used as a template for RT-qPCR with SYBR Green Supermix (Thermo Fisher Scientific, CA, USA) and the Light Cycler® 96 System (Roche, CA, USA). All RT-qPCR reactions were performed using biological (n = 10) and technical triplicates. The housekeeping gene *18S* was used as a reference gene. The primer sequences for RT-qPCR are listed in table S3. The cycle threshold values (Ct) data were imported into Microsoft Excel for the relative gene expression analysis. Quantification was based on $2^{(-\Delta\Delta CT)}$ method and using 1-hour post-fertilization (hpf) for *tars2* temporal expression, skin for *tars2* in tissue-specific expression, and the control MO-injected group for *tars2* MO as well as mRNA-injected group for normalization.

Statistical analysis

Each experiment was performed three times independently. Data are presented as mean value ± standard deviation (SD). Statistical analysis was performed using GraphPad Prism version 9.3 (GraphPad Software, San Diego, CA, USA). In all analyses, the significance level was set to 0.05. The *p* value was determined by one-way analysis of variance (ANOVA) with Tukey's multiple comparisons test.

Supplementary Results

Analysis of amino-acid conservation of TARS2 residues impacted by disease-associated missense variations

The amino acid conservation of residues impacted by disease-associated missense variants is analyzed within a robust multiple sequence alignment of ThrRS protein sequences from archaea, bacteria and eukaryotes (See Figure S1). It shows that E440 is strictly conserved (100%), P155 and R684 are highly conserved except in archaea, and F323 is conserved in eukaryotes but variable in other species. In addition, R109 is mainly conserved (>80%) but only in eukaryotes; and the residue corresponding to L544 is preferentially non-polar (i.e. V/I/L/M/A/F). The other affected positions (R327, A337, E342, R346, R419, A714) are not conserved.

A similar variability of conservation of the affected positions is observed for the previously reported variants of TARS2, with residues strictly (D560, P613) or highly

conserved (R452, except in rodents that actually display a W), conserved in only eukaryotes (P282), physico-chemically conserved (E or D for E425), or not conserved (T157, S258, E715). Noteworthy, the vast majority of the so-far reported pathology-related variants identified within mitochondrial aaRSs affect positions mainly not conserved (for updated statistics, see www.misynpat.org[6]). Indeed, a strict conservation of residues is the hallmark of a strong selective pressure and would reflect a strong structural and/or functional role.

Analysis of the structural environment of the residues affected by missense variants

The human TARS2 is an homodimeric class II aaRS with the catalytic core made of three conserved motifs[7]. It possesses a catalytic and tRNA anticodon binding domain (Figure 3A, B), an editing domain to hydrolyze incorrectly charged amino acids[8], and an N-terminal TGS domain (named after "ThrRS, GTPase and SpoT proteins") of unknown function. All disease-related TARS2 variants are positioned in a schematic representation of the modular organization of the enzyme (Figure 3C-G). The identification of 12 new missense variants (in red) greatly extends the pattern of reports with descriptions in all constitutive domains of the enzyme.

The investigation of the structural environment of the residues affected by the missense variants is made using known crystallographic structures (*Staphylococcus* aureus ThrRS, PDB: 1NYR [9] (Figure 3A) and one monomer of *Escherichia coli* ThrRS in complex with $tRNA^{Thr}$, PDB: 1QF6 [10] (Figure 3B) and a 3D model of the human TARS2 (downloaded from www.misynat.org). The correspondence of the residues from the three enzymes is given in Figure 3H.

As for the previously described D560H and R452W variants, E440K and R346C affect residues near the catalytic core (Figure 3C). R346 is located close to catalytic motif 1 and interacts with the backbone of a nearby helix. Its mutation may thus create local rearrangements. E440 belongs to motif 2 and interacts with the threonyl-adenylate, strongly suggesting a catalytic impact for the variant E440K. A functional impact is also anticipated for R684Q, since the corresponding conserved amino acid (R609) was shown in *E. coli* to directly interact with the tRNA anticodon nucleotide U36 (Figure

3E)[10]. As for P613, R327 is situated at the interface between the anticodon binding and the catalytic domains (Figure 3F). The replacement of a positively charged by a negatively charged residue (R327Q) might engender repulsive local changes and alter the inter-domain communication. Similarly, L544 is non-polar and surrounded by hydrophobic residues (Figure 3G). Its change into F (L544F) might engender a staking interaction with the aromatic ring of Y483, thus creating a local distortion. As for the editing domain, none of the reported variants impact residues identified to perform the editing function[8] (in orange in Figure 3D), P155 is however at close vicinity and its replacement by a leucine (P155L) might create local rearrangements. Finally, the other variants described in the present study impact residues that are located at the dimer interface (F323C, A337, E342D), in a loop that points to the solvent (R419W, E425G), or in the TGS domain (R109Q) (these are not shown).

Although all these observations must be experimentally confirmed, they serve the purpose of illustrating the diversity of the possible molecular impacts of the pathology-related missense variants of TARS2.

***Zebrafish tars2* mRNA was strongly expressed in developing brain**

Zebrafish *tars2* gene has not been well-annotated in Ensembl (LO017852.1-201) but predicted full length cDNA sequence can be found in NCBI database (Accession number: XM_021466791) as well as amino acid sequence (Accession number: XP_021322466.1).

To investigate the expression level of *tars2* during zebrafish embryonic development, we performed RT-qPCR that showed *tars2* transcript is expressed at about the same level starting from 1-hour post-fertilization (hpf) up to 120 hpf (Figure S3A). Analysis of *tars2* mRNA expression in different adult tissues of zebrafish revealed a significant enrichment in brain, eye, heart, muscle, notochord and testis (Figure S3B). Zebrafish expression data is Our data is in agreement with the RNA expression data from the Genotype-Tissue Expression (GTEx) database, where TARS2 mRNA in humans is enriched in the cerebellum, testis and skeletal muscles.

We performed whole-mount *in situ* hybridization (WISH) to investigate the spatial and temporal expression of *tars2* mRNA during zebrafish embryonic development. WISH data show starting from 1 hpf onward maternally-supplied and ubiquitous

expression of *tars2* mRNA during early embryogenesis (Figure S3C, D). By 24 hpf, the expression is more prominent in the forebrain, the presumptive optic tectum (TeO), eye, and the trunk muscles (Figure S3E). At 72 hpf, the expression is abundant in the head and pectoral fin but decreased visibly in trunk muscles (Figure S3F, F'). By 120 hpf, *tars2* mRNA expression was further decreased throughout the embryo but showed brain and pectoral fin specificity with additional expression in the intestine (black arrowhead, Figure S3H, H'). In conclusion, RT-qPCR, and WISH data show that *tars2* mRNA was initially ubiquitously expressed but gradually became more prominent in the brain, eye, muscles, pectoral fin, and intestine. Our findings suggest distinct tissue requirements for Tars2 during development that strongly correlated with the pleiotropic symptoms observed in individuals carrying *TARS2* variants.

Supplementary Tables and Figures

Table S1
Characteristics *of TARS2* variant.

Table S2
Genetic, clinical, and neuroradiological features of TARS2 deficiency individuals.

Table S3
List of oligos used in this study.

Figure S1. TARS multiple sequence alignment. A multiple sequence alignment (MSA) of TARS sequences from 52 organisms, representative of bacterial, archaeal and eukaryotic phylogenetic diversity. Sequences were aligned using the MAFFT program. The MSA was generated inside the workbench ORDALIE (ORDered ALIgnment Information Explorer) and drawn in SnapGene software. The consensus sequence with a threshold of >70% is provided on the top of the alignment, and the sequence conservation is highlighted with gray bars. Amino acids are marked with color highlighting properties and conservation (following the ClustalX option embedded in SnapGene). The sequence nomenclature is: a T for the Threonine specificity of the aaRS, followed by four letters indicating the Bacterial (bact), Archaeal (arch), or Eukaryotic cellular location (cyto for cytosolic, and mito for mitochondria), then an underscore followed by two four-letter codes for the Genus and species separated by a dot (e.g. Tmito_Homo.sapi stands for human TARS2). Missense variants described in the present study are indicated in red on the top of the alignment, those previously reported (see Figure 1 and Table S2 for corresponding references) are indicated in black.

Figure S2. *TARS2* c.387+6T>C leads to RNA transcripts lacking exons 3 and 4 containing a portion of the B-terminal editing domain. (A) Lymphoblastoid cell lines isolated from the proband, father and mother were treated +/– puromycin (lanes 1 and 2), +/- cycloheximide (lanes 3 and 4), or nothing (lane 5) and tested in duplicate. PCR primers were designed to amplify exons encompassing those predicted to be spliced by *in silico* prediction tools. Cells from the proband and mother carrying the c.387+6T>C variant produced RNA transcripts that resulted in PCR products ~250bp smaller than the wild type 700bp PCR product regardless of treatment. This reduction in size is equal to the loss of *TARS2* exons 3 and 4. The alternative splice product was not observed in the father nor in blood (bld), breast (brst), or colon (cln) samples, but was detected in brain (brn), suggesting it may be a tissue specific isoform. **(B)** CloneSeq on these same samples was performed as previously described. Relative quantification of CloneSeq results is shown as the percent spliced, confirming the presence of transcripts lacking *TARS2* exons 3 and 4 in the proband and mother, but not the father.

Figure S3. Detection of *tars2* mRNA expression in zebrafish. (A) RT-qPCR of *tars2* mRNA expression from 1 to 120 hpf in WT animals. The *tars2* mRNA expression can be detected starting from 18 hpf and then increased throughout development. Data shown are mean ± SD and compared to 18 hpf. **(B)** RT-qPCR of *tars2* mRNA expression in different adult tissues. Data shown are mean ± SD and compared to skin. (C-G') WISH was performed for detecting *tars2* expression during zebrafish embryogenesis. Each embryonic stage is shown at the top right of the panels (hpf, hours post-fertilization). **(C)** *tars2* expression measured by maternal mRNA. Dorsal view. **(D)** An 18 hpf embryo shown in lateral view, anterior to the left. **(E)** A 24 hpf embryo, flat mounted and shown in dorsal view (anterior to the left). fb: forebrain, mb: midbrain, hb: hindbrain, MHB midbrain and hindbrain boundary, TeO: optic tectum. A 72 hpf embryo shown in lateral view **(F)** and dorsal view **(F')** A 120 hpf embryo is shown in lateral view **(G)** and dorsal view **(G').** Arrowheads indicate the intestine. Scale bar = 250 μm.

Figure S4. RT-PCR analysis of *tars2* splice-blocking morpholino (MO). (A) Schematic representation of partial *tars2* pre-mRNA. The MO was designed targeting exon 11/ intron11, as were primer binding sites used for RT-PCR. **(B)** mRNA from uninjected, control morphants and *tars2* morphants was used as a template to amplify sequence. Both uninjected and ctrl MO samples showed a single band (size around 500 bp) as indicated by the black arrow. *tars2* morphants showed bands of multiple different size (asterisk), indicating aberrant spliced mRNAs produced after MO injection.

Figure S5. Gene expression analysis in *tars2* morphants. (A-F) Reverse transcription quantitative polymerase chain reaction (RT-qPCR) examination in ctrl MO, *tars2* MO and *tars2* MO rescue embryos at 3 dpf. The expression levels were normalized to the *18S* housekeeping gene. Error bars = mean ± SD. One-way ANOVA with Tukey's multiple comparisons correction: ns, not significant $p \geq 0.05$, *$p < 0.05$, **$p < 0.01$, ***$p < 0.001$ and ****$p < 0.0001$ compared to *tars2* MO group.

Figure S6. Functional validation of human *TARS2* variants in zebrafish.

Quantification of the head size of ctrl MO, or *tars2* morphants and morphants rescued with either human wild-type (WT) or different variants, as labeled. The size measurements were calculated as the percentage of the mean value of ctrl MO embryos. n = 30 embryos. Error bars = mean ± SD. The statistical significances were calculated by one-way ANOVA with Tukey's multiple comparisons test: ns, not significant $p \geq 0.05$, $^{*}p < 0.05$, $^{**}p < 0.01$, $^{***}p < 0.001$, and $^{****}p < 0.0001$ were compared to *tars2* MO group and displayed in blue text. ns, not significant $p \geq 0.05$, $^{\#}p < 0.05$, $^{\#\#}p < 0.01$, $^{\#\#\#}p < 0.001$, and $^{\#\#\#\#}p < 0.0001$ were compared to the uninjected control group and displayed in red text.

Supplementary References

1. Katoh K, Standley DM. MAFFT multiple sequence alignment software version 7: improvements in performance and usability. Mol Biol Evol. 2013;30(4):772-780.
2. Thisse C, Thisse B. High-resolution in situ hybridization to whole-mount zebrafish embryos. Nat Protoc. 2008;3(1):59-69.
3. Lin SJ, Vona B, Barbalho PG, et al. Biallelic variants in KARS1 are associated with neurodevelopmental disorders and hearing loss recapitulated by the knockout zebrafish. Genet Med. 2021;23(10):1933-1943.
4. Stainier DYR, Raz E, Lawson ND, et al. Guidelines for morpholino use in zebrafish. PLoS Genet. 2017;13(10):e1007000.
5. Tucker B, Lardelli M. A rapid apoptosis assay measuring relative acridine orange fluorescence in zebrafish embryos. Zebrafish. 2007;4(2):113-116.
6. Sissler M, Gonzalez-Serrano LE, Westhof E. Recent Advances in Mitochondrial Aminoacyl-tRNA Synthetases and Disease. Trends Mol Med. 2017;23(8):693-708.
7. Cusack S, Berthet-Colominas C, Hartlein M, Nassar N, Leberman R. A second class of synthetase structure revealed by X-ray analysis of Escherichia coli seryl-tRNA synthetase at 2.5 A. Nature. 1990;347(6290):249-255.
8. Dock-Bregeon A, Sankaranarayanan R, Romby P, et al. Transfer RNA-mediated editing in threonyl-tRNA synthetase. The class II solution to the double discrimination problem. Cell. 2000;103(6):877-884.
9. Torres-Larios A, Sankaranarayanan R, Rees B, Dock-Bregeon AC, Moras D. Conformational movements and cooperativity upon amino acid, ATP and tRNA binding in threonyl-tRNA synthetase. J Mol Biol. 2003;331(1):201-211.
10. Sankaranarayanan R, Dock-Bregeon AC, Romby P, et al. The structure of threonyl-tRNA synthetase-tRNA(Thr) complex enlightens its repressor activity and reveals an essential zinc ion in the active site. Cell. 1999;97(3):371-381.

Figure S1

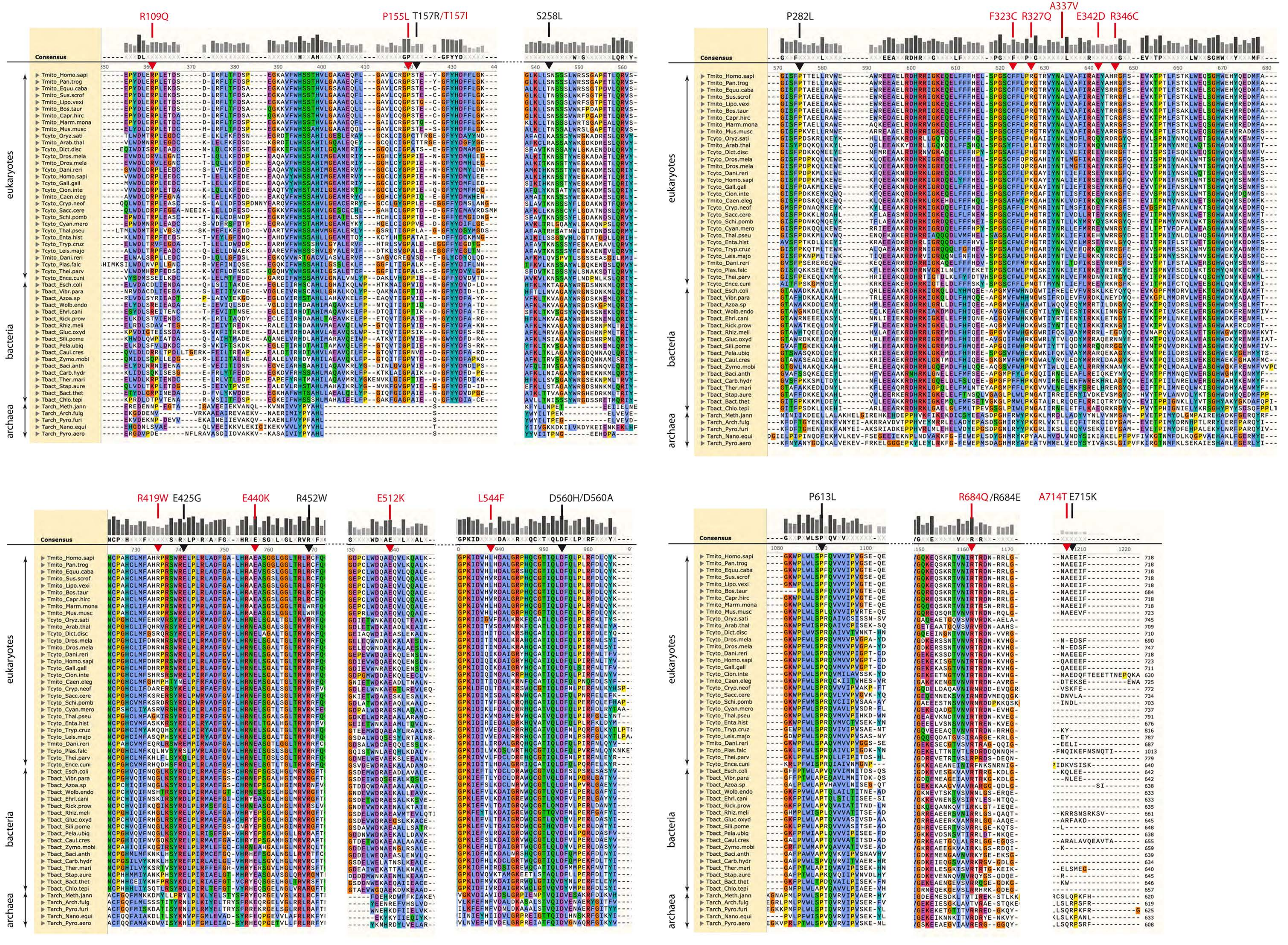

R109Q
P155L
T157R/T157I
S258L
P282L
F323C
R327Q
A337V
E342D
R346C
R419W
E425G
E440K
R452W
E512K
L544F
D560H/D560A
P613L
R684Q/R684E
A714T
E715K
Consensus
eukaryotes
bacteria
archaea

Figure S2

A

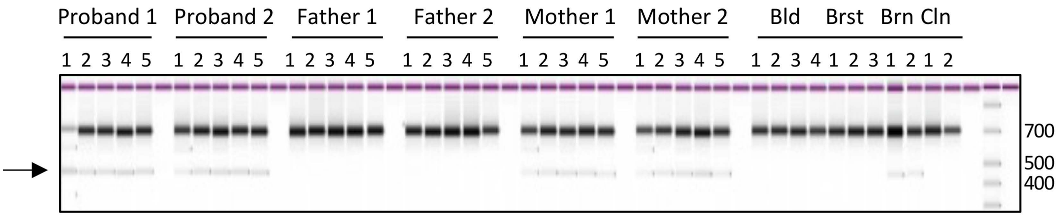
Proband 1
Proband 2
Father 1
Father 2
Mother 1
Mother 2
Bld
Brst
Brn Cln
1 2 3 4 5 1 2 3 4 5 1 2 3 4 5 1 2 3 4 5 1 2 3 4 5 1 2 3 4 5 1 2 3 4 1 2 3 1 2 1 2
700
500
400

B

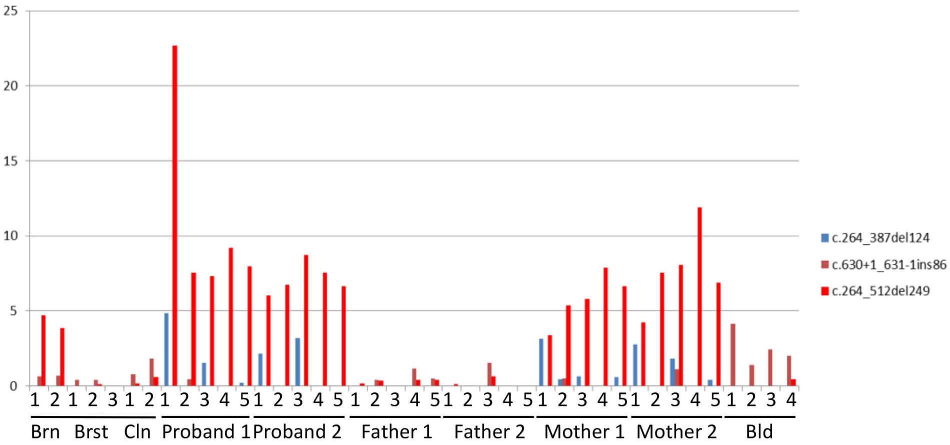
25
20
15
10
5
0
c.264_387del124
c.630+1_631-1ins86
c.264_512del249
1 2 1 2 3 1 2 1 2 3 4 5 1 2 3 4 5 1 2 3 4 5 1 2 3 4 5 1 2 3 4 5 1 2 3 4 5 1 2 3 4
Brn
Brst
Cln
Proband 1
Proband 2
Father 1
Father 2
Mother 1
Mother 2
Bld

Figure S3

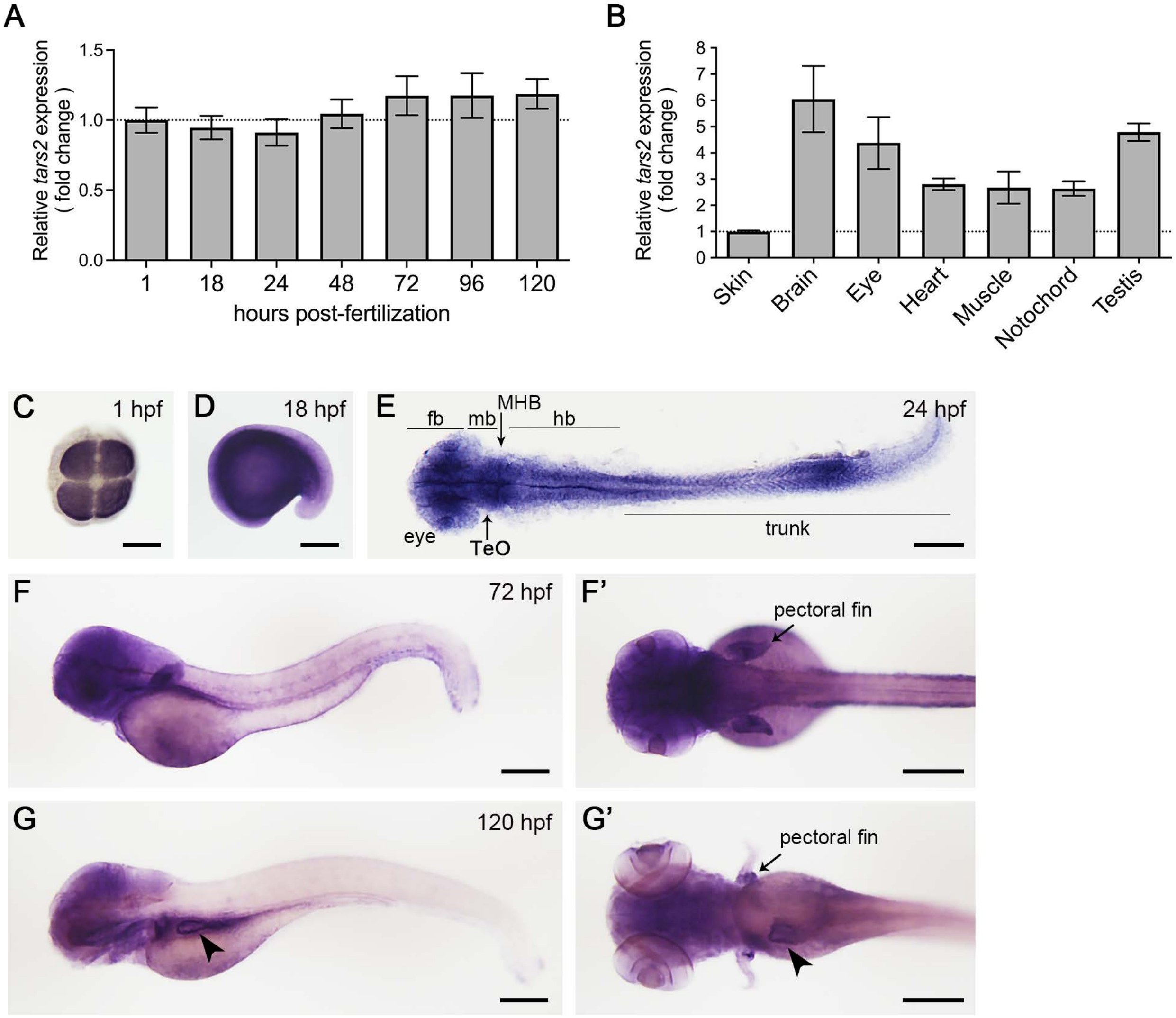
A
Relative tars2 expression (fold change)
1.5
1.0
0.5
0.0
1
18
24
48
72
96
120
hours post-fertilization
B
Relative tars2 expression (fold change)
8
7
6
5
4
3
2
1
0
Skin
Brain
Eye
Heart
Muscle
Notochord
Testis
C
1 hpf
D
18 hpf
E
24 hpf
fb
mb
MHB
hb
eye
TeO
trunk
F
72 hpf
F'
pectoral fin
G
120 hpf
G'
pectoral fin

Figure S4

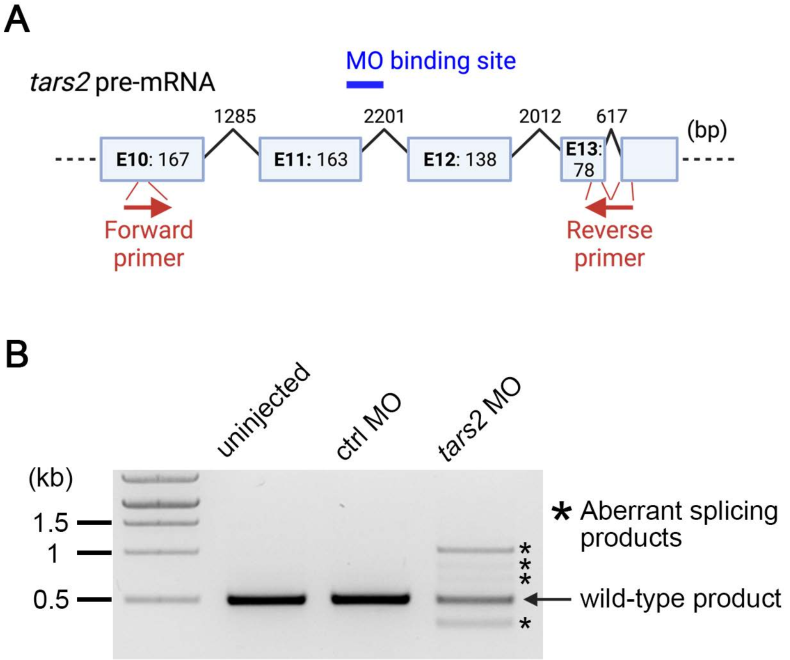
A
tars2 pre-mRNA
MO binding site
1285
2201
2012
617
(bp)
E10: 167
E11: 163
E12: 138
E13: 78
Forward primer
Reverse primer
B
uninjected
ctrl MO
tars2 MO
(kb)
1.5
1
0.5
* Aberrant splicing products
wild-type product

Figure S5

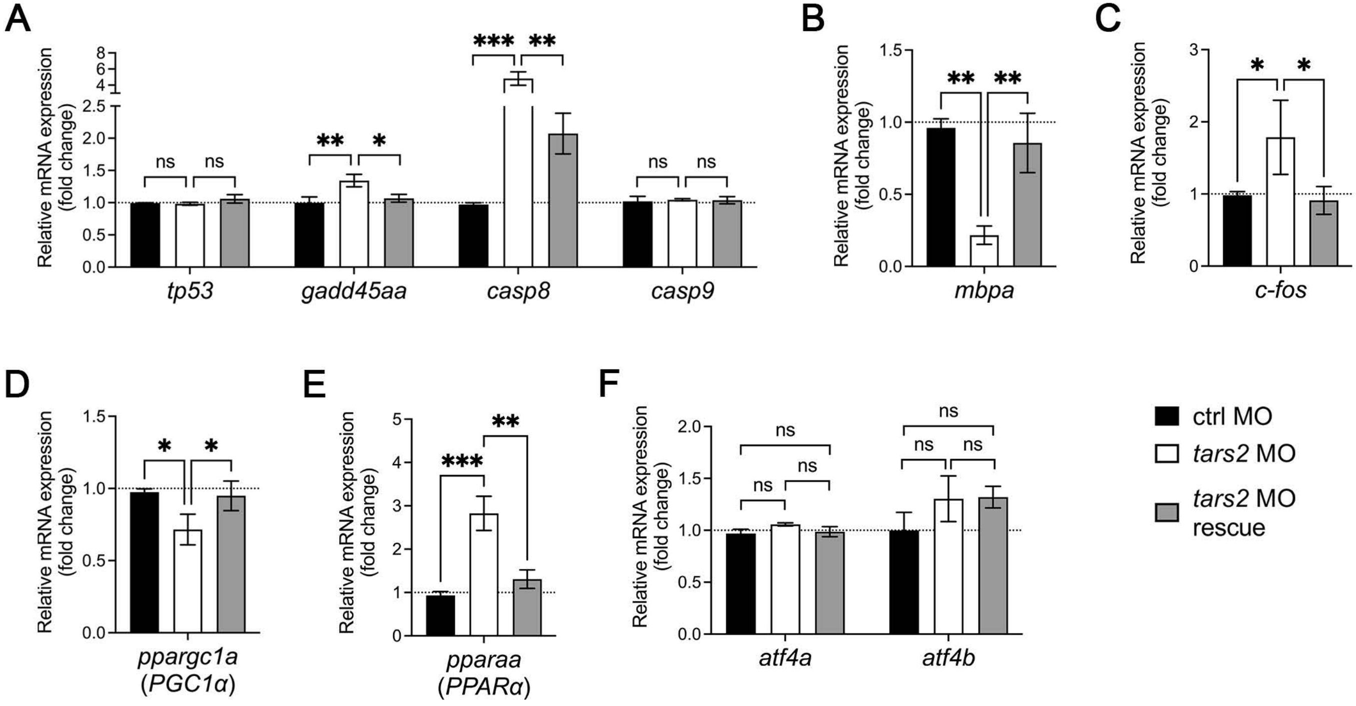
A
B
C
D
E
F
Relative mRNA expression (fold change)
tp53
gadd45aa
casp8
casp9
mbpa
c-fos
ppargc1a (PGC1α)
pparaa (PPARα)
atf4a
atf4b
ns
**
*
***
ctrl MO
tars2 MO
tars2 MO rescue

Figure S6

Head size measurments of MO co-injected with human TARS2 variant(s)

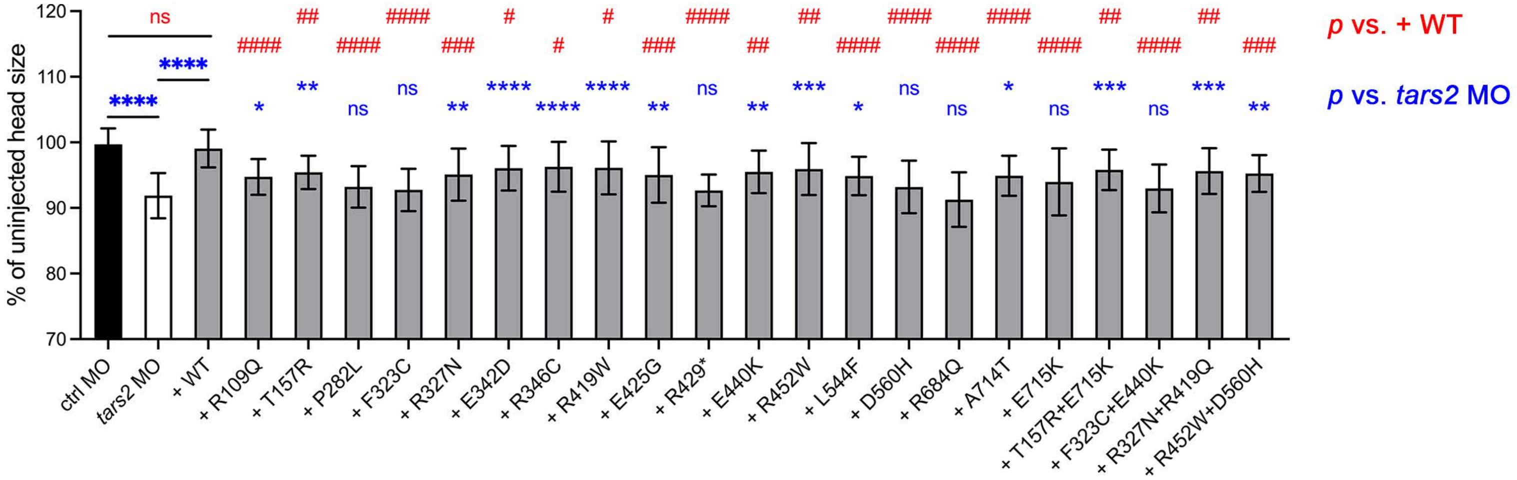

Supplement: Supplementary Information [file mmc1.pdf]
